# Supplementary material for: How do high ambient temperatures affect infant feeding practices? A prospective cohort study of postpartum women in Bobo-Dioulasso, Burkina Faso
Source: BMJ Open. 2022 Oct 5;12(10):e061297. doi: 10.1136/bmjopen-2022-061297 (PMC9535177; doi:10.1136/bmjopen-2022-061297)
Supplement: Supplementary data [file bmjopen-2022-061297supp001.pdf]

SUPPLEMENTAL MATERIAL

How do high ambient temperatures affect infant feeding practices? A prospective cohort study of postpartum women in Bobo-Dioulasso, Burkina Faso

Contents

Table S1. Outcomes, exposure, potential confounders and covariates considered in all models. 2

Figure S1. Daily maximum and minimum temperatures and seasons in Bobo-Dioulasso, Burkina Faso 3

Table S2. Effect estimates, 95% confidence intervals, and *p*-values of each variable included in the multilevel linear model of temperature-breastfeeding association 4

Table S3. Effect estimates, 95% confidence intervals, and *p*-values of each variable included in the multilevel linear model of temperature-childcare association 5

References 5

Part C, *et al. BMJ Open* 2022; 12:e061297. doi: 10.1136/bmjopen-2022-061297

Table S1. Outcomes, exposure, potential confounders and covariates considered in all models.

Variable	Definition	Units	Type	Model
**Outcomes**				
Breastfeeding duration	Time spent breastfeeding infants aged 4+ days	Minutes per day	Continuous	MLM (BF)
Exclusive breastfeeding	No liquids other than breast milk given in past 24h (infants aged <6 months)	Yes, No	Binary	Logistic (EBF)
Supplementary feeding	Any liquid other than breast milk given in past 24h (infants aged 6-12 months)	Yes, No	Binary	Logistic (SF)
Childcare duration	Time spent caring for children (bathing/dressing, playing/watching, tending to when unwell, etc.)	Minutes per day	Continuous	MLM (CH)
**Exposure**				
Daily mean temperature	Mean temperature in Bobo-Dioulasso on day before interview	°C	Continuous	All
**Confounders**				
Season/time trends	Seasonal patterns (unrelated to temperature) and long-term trends	Month of interview	Categorical	All
Interview round	Interview (baseline, 3 months post-baseline, 9 months post-baseline)	1, 2, 3	Categorical	BF, CH
Infant age	Date of interview – Date of delivery	Weeks	Continuous	All
Income-generating work	Time spent on professional or educational activities	Minutes per day	Continuous	All
Domestic work	Time spent on domestic activities (excluding family care)	Minutes per day	Continuous	BF, EBF, SF
**Covariates**				
Number born	Singleton or multiple birth	1, 2	Categorical	All
Gravidity	Total number of pregnancies, including current	1, 2–5, 6+ pregnancies	Categorical	All
Maternal age	Age of mother at baseline	≤ 19, 20–34, ≥ 35 years	Categorical	All
Living arrangements	Mothers' living arrangements with partner at each interview	With partner full-time, With partner periodically, Not with partner, Not in a relationship	Categorical	CH
Residential area	Mother's area of residence	Urban, Rural	Categorical	All
Roofing materials	Roofing materials of mother's house	Natural, Rudimentary, Contemporary	Categorical	All
**Interactions**				
*Temperature * Residential area*	Daily mean temperature and mother's area of residence	°C * Urban/Rural	Interaction	All
*Temperature * Infant age*	Daily mean temperature and infant age	°C * Weeks	Interaction	All
*Temperature * Roofing materials*	Daily mean temperature and roofing materials of mother's house	°C * Natural/Rudimentary/ Contemporary	Interaction	All

MLM = Multilevel model. BF = Breastfeeding duration. CH = Childcare duration. SF = Supplementary feeding. EBF = Exclusive breastfeeding.

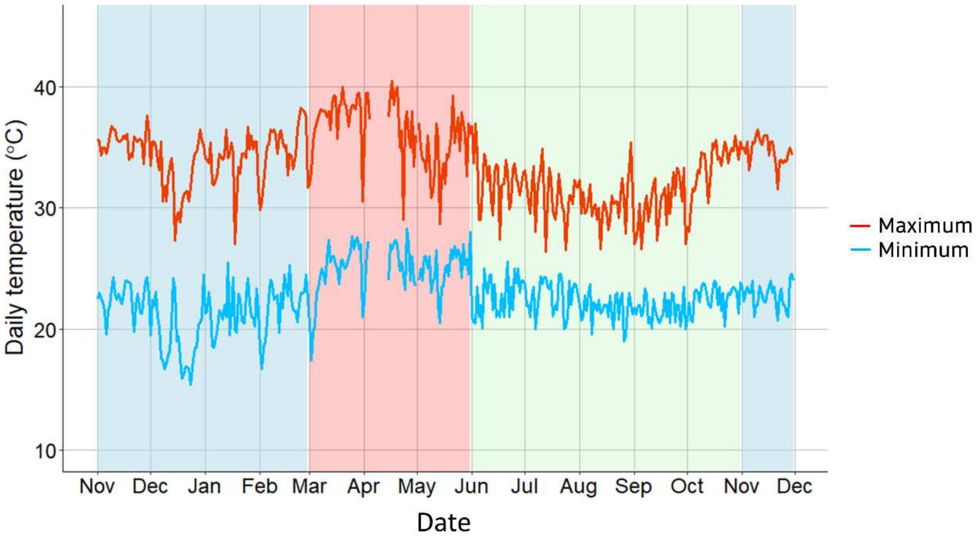

Figure S1. Daily maximum and minimum temperatures and seasons in Bobo-Dioulasso, Burkina Faso, between 1st Nov 2013 and 30th Nov 2014. Blue = dry, cooler season (November–February), red = dry, hot season (March–May), green = rainy season (June–October). Data source: TuTiempo.net [1].

Table S2. Effect estimates, 95% confidence intervals, and *p*-values of each variable included in the autoregressive multilevel linear model for the exposure-response association between temperature and breastfeeding duration (minutes/day).

Variable	Estimate	95% CI		*p*-value
Daily mean temperature (°C)	-2.29	-4.63	0.04	0.05
Interview round (reference: 1)				
2	32.01	-52.53	116.54	0.46
3	135.46	59.35	211.57	<0.001
Month of data collection (reference: January)				
February	-34.16	-61.25	-7.07	0.01
March	-61.79	-89.14	-34.45	<0.001
April	-7.70	-95.20	79.79	0.86
May	-22.75	-106.68	61.18	0.59
June	-37.01	-122.13	48.12	0.39
July	-37.57	-135.16	60.02	0.45
September	-89.72	-167.34	-12.09	0.02
October	-102.25	-178.37	-26.14	<0.01
November	-66.34	-136.35	3.66	0.06
December	-13.72	-35.25	7.81	0.21
Singleton/multiple birth (reference: singleton)				
Multiple birth	70.46	48.43	92.49	< 0.001
Residential area (reference: rural)				
Urban	18.26	-5.76	42.29	0.13
Income-generating activities (minutes/day)	0.02	0.00	0.04	0.02

Part C, *et al. BMJ Open* 2022; 12:e061297. doi: 10.1136/bmjopen-2022-061297

Table S3. Effect estimates, 95% confidence intervals, and *p*-values of each variable included in the autoregressive multilevel linear model for the exposure-response association between temperature and childcare duration (minutes/day).

Variable	Estimate	95% CI		*p*-value
Daily mean temperature (°C)	0.65	0.06	1.24	0.03
Interview round (reference: 1)				
2	-8.09	-34.64	18.47	0.55
3	-15.25	-36.13	5.63	0.15
Month of data collection (reference: January)				
February	-1.75	-7.64	4.13	0.56
March	-4.36	-10.33	1.60	0.15
April	9.96	-17.18	37.09	0.47
May	8.99	-17.90	35.87	0.51
June	8.32	-18.84	35.48	0.55
July	3.48	-25.75	32.71	0.82
September	15.08	-5.95	36.10	0.16
October	12.88	-8.13	33.90	0.23
November	16.52	-2.63	35.66	0.09
December	14.34	8.75	19.92	< 0.001
Singleton/multiple birth (reference: singleton)				
Multiple birth	17.43	11.53	23.33	< 0.001
Infant age (weeks)	0.09	-0.04	0.21	0.17
Maternal age (reference: 20-34 years)				
< 19 years	-5.68	-8.68	-2.69	< 0.001
≥ 35 years	0.29	-2.39	2.97	0.83
Income-generating activities (minutes/day)	-0.01	-0.01	-0.00	0.04
Living arrangements (reference: Lives with partner full-time)				
Lives with partner periodically	-1.91	-5.73	1.92	0.33
Does not live with partner	-2.57	-7.42	2.29	0.30
Not in a relationship	-8.68	-12.47	-4.89	< 0.001

References

1 TuTiempo. Clima Bobo-Dioulasso. Datos climáticos: 1973 - 2021. https://www.tutiempo.net/clima/ws-655100.html.

Part C, *et al. BMJ Open* 2022; 12:e061297. doi: 10.1136/bmjopen-2022-061297
