# Supplementary material for: How do high ambient temperatures affect infant feeding practices? A prospective cohort study of postpartum women in Bobo-Dioulasso, Burkina Faso
Source: BMJ Open. 2022 Oct 5;12(10):e061297. doi: 10.1136/bmjopen-2022-061297 (PMC9535177; doi:10.1136/bmjopen-2022-061297)
Supplement: Author's manuscript [file bmjopen-2022-061297.draft_revisions.pdf]

BMJ Open is committed to open peer review. As part of this commitment we make the peer review history of every article we publish publicly available.

When an article is published we post the peer reviewers' comments and the authors' responses online. We also post the versions of the paper that were used during peer review. These are the versions that the peer review comments apply to.

The versions of the paper that follow are the versions that were submitted during the peer review process. They are not the versions of record or the final published versions. They should not be cited or distributed as the published version of this manuscript.

BMJ Open is an open access journal and the full, final, typeset and author-corrected version of record of the manuscript is available on our site with no access controls, subscription charges or pay-per-view fees (http://bmjopen.bmj.com).

If you have any questions on BMJ Open's open peer review process please email info.bmjopen@bmj.com

BMJ Open

How do high ambient temperatures affect infant feeding practices? A prospective cohort study of postpartum women in Bobo-Dioulasso, Burkina Faso

Journal:	*BMJ Open*
Manuscript ID	bmjopen-2022-061297
Article Type:	Original research
Date Submitted by the Author:	21-Jan-2022

Complete List of Authors:	Part, Chérie; London School of Hygiene & Tropical Medicine, Department of Public Health, Environments and Society<br>Filippi, Veronique; London School of Hygiene & Tropical Medicine, Department of Infectious Disease Epidemiology<br>Cresswell, Jenny; London School of Hygiene & Tropical Medicine, Department of Infectious Disease Epidemiology<br>Ganaba, Rasmané; AFRICSanté<br>Hajat, Shakoor; London School of Hygiene & Tropical Medicine, Department of Public Health, Environments and Society<br>Nakstad, Britt; University of Oslo, Institute of Clinical Medicine; University of Botswana, Department of Pediatrics and Adolescent Health<br>Roos, Nathalie; Karolinska Institutet, Department of Medicine<br>Kadio, Kadidiatou; Institut de Recherche en Sciences de la Santé<br>Chersich, Matthew; University of the Witwatersrand, Wits Reproductive Health and HIV Institute<br>Lusambili, Adelaide; Aga Khan University - Kenya, Department of Population Health<br>Kouanda, Seni; Institut de Recherche en Sciences de la Sante<br>Kovats, Sari; London School of Hygiene & Tropical Medicine, Department of Public Health, Environments and Society

Keywords:	EPIDEMIOLOGY, NUTRITION & DIETETICS, Community child health < PAEDIATRICS, PUBLIC HEALTH

SCHOLARONE™
Manuscripts

I, the Submitting Author has the right to grant and does grant on behalf of all authors of the Work (as defined in the below author licence), an exclusive licence and/or a non-exclusive licence for contributions from authors who are: i) UK Crown employees; ii) where BMJ has agreed a CC-BY licence shall apply, and/or iii) in accordance with the terms applicable for US Federal Government officers or employees acting as part of their official duties; on a worldwide, perpetual, irrevocable, royalty-free basis to BMJ Publishing Group Ltd ("BMJ") its licensees and where the relevant Journal is co-owned by BMJ to the co-owners of the Journal, to publish the Work in this journal and any other BMJ products and to exploit all rights, as set out in our licence.

The Submitting Author accepts and understands that any supply made under these terms is made by BMJ to the Submitting Author unless you are acting as an employee on behalf of your employer or a postgraduate student of an affiliated institution which is paying any applicable article publishing charge ("APC") for Open Access articles. Where the Submitting Author wishes to make the Work available on an Open Access basis (and intends to pay the relevant APC), the terms of reuse of such Open Access shall be governed by a Creative Commons licence – details of these licences and which Creative Commons licence will apply to this Work are set out in our licence referred to above.

Other than as permitted in any relevant BMJ Author's Self Archiving Policies, I confirm this Work has not been accepted for publication elsewhere, is not being considered for publication elsewhere and does not duplicate material already published. I confirm all authors consent to publication of this Work and authorise the granting of this licence.

For peer review only

HOW DO HIGH AMBIENT TEMPERATURES AFFECT INFANT FEEDING PRACTICES? A PROSPECTIVE COHORT STUDY OF POSTPARTUM WOMEN IN BOBO-DIOULASSO, BURKINA FASO

Chérie Part*[1], Véronique Filippi[2], Jenny A. Cresswell[2], Rasmané Ganaba[3], Shakoor Hajat[1], Britt Nakstad[4,5], Nathalie Roos[6], Kadidiatou Kadio[7], Matthew Chersich[8], Adelaide Lusambili[9], Seni Kouanda[7], Sari Kovats[1]

[1] Department of Public Health, Environments and Society, London School of Hygiene & Tropical Medicine, London, United Kingdom.

[2] Department of Infectious Disease Epidemiology, London School of Hygiene & Tropical Medicine, London, UK.

[3] AFRICSanté, Bobo-Dioulasso, Burkina Faso.

[4] Division of Child and Adolescent Health, Institute of Clinical Medicine, University of Oslo, Oslo, Norway

[5] Department of Pediatrics and Adolescent Health, University of Botswana, Gaborone, Botswana

[6] Department of Medicine, Clinical Epidemiology Division, Karolinska Institutet, Stockholm, Sweden

[7] Departement Biomédical et Santé Publique, Institut de Recherche en Sciences de la Santé, Ouagadougou, Burkina Faso.

[8] Wits Reproductive *Health* and HIV Institute, University of the Witwatersrand, Johannesburg, South Africa.

For peer review only - http://bmjopen.bmj.com/site/about/guidelines.xhtml

[9] Department of Population Health, Medical College, Aga Khan University, Nairobi, Kenya.

* Corresponding author email: cherie.part@lshtm.ac.uk. Address: Department of Public Health, Environments and Society, LSHTM, 15-17 Tavistock Place, London, WC1H 9SH.

For peer review only

For peer review only - http://bmjopen.bmj.com/site/about/guidelines.xhtml

ABSTRACT

Objective: To examine the effects of high ambient temperature on infant feeding practices and childcare.

Design: Secondary analysis of quantitative data from a prospective cohort study.

Setting: Community-based interviews in the commune of Bobo-Dioulasso, Burkina Faso. Exclusive breastfeeding is not widely practiced in Burkina Faso.

Participants: 866 women were interviewed over a 12-month period, with a 1:1 ratio of urban:rural residence. Participants were between 20 weeks gestation and 22 weeks postpartum at recruitment. Retention at the third (final) interview was 90%.

Exposure: Daily mean temperature (°C) measured at one weather station in Bobo-Dioulasso. Meteorological data were obtained from publicly available archives.

Primary outcome measures: Self-reported time spent breastfeeding (minutes/day); exclusive breastfeeding of infants under six months (no fluids other than breast milk provided in past 24 hours); supplementary feeding of infants under 12 months (any fluid other than breast milk provided in past 24 hours); time spent caring for children (minutes/day).

Results: The population experienced year-round high temperatures (intra-annual range in daily mean temperature 22.6–33.7°C). Breastfeeding decreased by 2.3 minutes/day (95% CI -4.6 to 0.04, p=0.05), and childcare increased by 0.6 minutes/day (0.06 to 1.2, p=0.03), with every 1°C increase in same-day mean temperature. Temperature interacted with infant age to affect breastfeeding duration (p=0.02), with a stronger (negative) association between temperature and time spent breastfeeding as infants aged (4 days–57 weeks). There was no

For peer review only

For peer review only - http://bmjopen.bmj.com/site/about/guidelines.xhtml

46 strong evidence of association between temperature and exclusive breastfeeding or supplementary feeding.

Conclusions: Women spend considerably less time breastfeeding (~25 minutes/day) during the hottest, compared to coolest, times of the year. Climate change adaptation plans for health should include advice to breastfeeding mothers during periods of high temperature.

For peer review only

For peer review only - http://bmjopen.bmj.com/site/about/guidelines.xhtml

Strengths and limitations of this study

- This is the first study to quantify acute effects of ambient heat on breastfeeding behaviour, with extensive confounder control and sensitivity analysis.
- Multi-stage stratified sampling was used to select a population-representative cohort of pregnant and postpartum women in the commune of Bobo-Dioulasso, Burkina Faso.
- Outcome measures relied on self-reports; however questions were embedded within an extensive interview schedule, reducing the likelihood of response bias.
- The small sample size and short recruitment window may have limited our ability to detect statistically significant associations.

For peer review only

INTRODUCTION

Climate change is a growing threat to population health in Africa,[1, 2] with heatwaves increasing in severity and duration, especially in the Sahel.[3] Maternal and neonatal health will be affected[4] through the adverse effects of heat on preterm birth,[5] stillbirth,[5] and maternal nutrition,[6] Mothers report difficulties with breastfeeding during periods of high temperature[7] and some women perceive breast milk as insufficient to hydrate babies in hot weather, causing mothers to supplement breast milk with water.[8-10]

Breastfeeding and, in particular, exclusive breastfeeding has well-established benefits for child health and development.[11-13] Breastfeeding reduces the risk of diarrhoea and respiratory infections among infants, and is associated with a higher intelligence quotient and reduced obesity in later life.[13] There are also benefits for maternal health, with nursing mothers at lower risk of breast and, potentially, ovarian cancers.[13] The World Health Organization recommends that infants are fed with breastmilk exclusively for the first six months and that no solids or other liquids are given during this period, including water.[14] However, the self-reported prevalence of exclusive breastfeeding is low in many African countries.[13, 15] In Burkina Faso, less than 25% of women reported exclusive breastfeeding of their young infants (less than six months old) in 2010-2015.[15]

Numerous factors influence infant feeding practices in Africa, including the competing time demands of women's domestic and agricultural workloads,[8, 16-18] which vary with season and weather conditions in rural settings,[19] In rural Ethiopia, infants were less likely to be exclusively breastfed for the recommended six months if rainfall was high during the primary agricultural season as demand for agricultural labour among nursing mothers increased.[19] In Burkina Faso, women work to supplement household income (particularly in agriculture, horticulture, and small trade) as well as undertaking important domestic responsibilities

For peer review only - http://bmjopen.bmj.com/site/about/guidelines.xhtml

(including gathering food, water, fuel, and feeding livestock).[6] Most women work in the informal sector.[20] Therefore, paid maternity leave is uncommon and many women return to work early in the postpartum period.

Average monthly temperatures in Burkina Faso range between 25–33°C[21] and the impacts on infant feeding practices are largely unknown. Several studies report seasonal patterns in breastfeeding behaviour in other regions of Africa,[22-24] South America,[25] and South Asia.[26] An older study in rural Egypt reported a higher incidence of prelacteal feeding in warmer months, leading to a reduction in exclusive breastfeeding in succeeding weeks and an increased incidence of diarrhoea in the first year of life.[27] However, such studies are insufficient to demonstrate an effect of ambient temperature as demands on mothers and other potentially important drivers (e.g. household food security) also vary seasonally. With daily temperatures in West Africa expected to exceed 50°C in some regions,[28] such research is essential so that maternal and child health programmes can be updated.

This study aims to examine the effects of outdoor temperature on infant feeding practices and childcare provided by postpartum women in western Burkina Faso. We hypothesised that: (a) Time spent breastfeeding is associated with same-day temperature (women may either increase breastfeeding due to perceived dehydration or decrease breastfeeding due to increased discomfort); (b) Women are less likely to breastfeed exclusively as temperatures rise; (c) Women are more likely to provide supplementary fluids as temperatures rise; (d) Time spent caring for children is associated with temperature.

METHODS

We undertook secondary analyses of quantitative data from an observational prospective cohort study of pregnant and postpartum women in Bobo-Dioulasso, Burkina Faso (the PopDev study), which aimed to assess the impacts of pregnancy on income- and non-income-

For peer review only - http://bmjopen.bmj.com/site/about/guidelines.xhtml

generating activities among women in Burkina Faso, and to identify interventions to increase household income.[29]

Participants

Multi-stage stratified (urban vs. rural) sampling was used to select a population-representative cohort of pregnant and postpartum women (female sex) in the commune of Bobo-Dioulasso. The 2006 census was used as the sampling frame. Participants were sampled from 38 locality clusters (14 urban, 24 rural). Women were eligible to participate in the PopDev study if aged 15-45 years and between seven months gestation and three months postpartum at recruitment.[29] Sixty-two women did not meet the criteria for PopDev, but were retained in the dataset for secondary analyses. The dataset used herein comprised 866 women aged 14-47 years who were between 20 weeks gestation and 22 weeks postpartum at baseline.

Setting

The commune of Bobo-Dioulasso is predominantly urban, with small agricultural settlements and villages located in rural areas around the urban centre. The commune includes the second largest city in Burkina Faso (Bobo-Dioulasso) with approximately 900,000 inhabitants.[30]

The commune has a tropical savannah climate,[31] with two distinct seasons; dry (November–May) and rainy (June–October). During the dry season, average temperatures are highest in March–May and lower in November–February.

Data collection

Participants were interviewed in their homes at three time points: cohort entry, and three and nine months thereafter. Retention at the nine-month visit was 90%. Several attempts were

For peer review only

made to interview each woman at each interview round. The reason for non-interview was recorded, when possible. Eight hundred and 39 participants were recruited immediately before/during the first round of interviews (29 November 2013 – 23 March 2014). Twenty-seven women were recruited during the second or third round of interviews (04 March 2014 – 09 September 2014; 02 September 2014 – 12 November 2014, respectively). All interviews occurred between 29 November 2013 and 12 November 2014, using structured questionnaires to ensure the same wording of questions for all participants. All questions were designed to be non-leading.

During each interview, participants were asked to recall their activities on the previous day (or two days previous when the day before interview was atypical). Using an exhaustive list of activities, women were asked which activities they carried out and how many minutes were spent on each activity. Breastfeeding, caring for children, income-generating work, attending classes, and household chores were included in this list.

At first interview following childbirth, participants were asked how many children were born and the date of delivery. At interviews two and three, women were asked if they were still breastfeeding and which, if any, additional fluids had been given to their baby/babies in the past 24 hours. Questionnaires are available at: https://datacompass.lshtm.ac.uk/id/eprint/64/.

Meteorological data

Daily meteorological data (mean, minimum and maximum temperature [°C], relative humidity [%], and windspeed [km/h]) were obtained from TuTiempo.net[32] for a single weather station in Bobo-Dioulasso, located in the industrial district (Zone Industrielle) (11°09'36.0"N 4°18'36.0"W). Eleven days of temperature data were missing over the study period and were excluded from the analysis.

For peer review only - http://bmjopen.bmj.com/site/about/guidelines.xhtml

Data analyses

All outcomes, exposure, potential confounding variables, and covariates are defined in table S1 (supplementary material). Key outcomes (self-reported breastfeeding duration, exclusive breastfeeding, supplementary feeding, and childcare duration on the day before interview) were examined in relation to daily mean temperature on the day before interview. Daily mean temperature correlated strongly with daily minimum (r=0.8, p<0.001) and maximum temperatures (r=0.89, p<0.001) and was considered the best approximation of overall exposure during the recall period.

The functional form of temperature-time use (breastfeeding, childcare) associations were determined by: (i) aggregating outcome data to daily level, (ii) fitting natural cubic splines of time to each outcome series (to adjust for seasonal patterns and trends unrelated to temperature), and (iii) examining locally-weighted smoothing of Pearson's standardised residuals from the fitted splines, plotted against daily mean temperature.

Multilevel linear regression was used to estimate the effects of daily mean temperature on time spent (i) breastfeeding, and (ii) caring for children. Interview contacts (level one) were nested within individual participants (level two), nested within the locality clusters from which the population was sampled (level three). Each level was defined as a random coefficient with random intercept.

Supplementary feeding and exclusive breastfeeding data were only available for interviews two and three. Data were restricted to interview two in order to reduce model complexity and to focus on the hottest months of the year. Associations with daily mean temperature were initially assessed by comparing a subset of women interviewed following one of the hottest days between March and June 2014 (>90th percentile of daily mean temperature) with a subset of women interviewed following one of the coolest days between March and June

For peer review only

For peer review only - http://bmjopen.bmj.com/site/about/guidelines.xhtml

2014 (<10th percentile of daily mean temperature). Logistic regression was then used to test for associations between mean temperature and the odds of (i) exclusive breastfeeding and (ii) supplementary feeding, adjusting for important confounders and covariates (table S1).

Separate models (either multilevel or logistic) were developed for each outcome following a forward stepwise process. Dummy variables adjusted for interview round (multilevel models only) and month of data collection. Adjusting for month (rather than season) of interview provided tighter control of possible confounders, such as household food security and fasting during Ramadan. Other covariates were retained in the model if they were significantly associated with the outcome ($p<0.05$), improved model fit (reduced the Akaike Information Criterion by ≥2%), and/or changed the temperature effect by ≥10%. Cases with missing data were excluded from the analysis. Participants lost to follow-up were included in the analysis.

Interactions between mean temperature and: (i) infant age; (ii) urban/rural residence; and (iii) roofing materials of the home were tested in all models. Final multilevel models were specified with a first-order autoregressive correlation structure, allowing for unequal spacing of interviews.

Sensitivity analyses involved re-specifying models with: (i) alternative levels of seasonal control (dummy variables for 'season' rather than 'month'; natural cubic splines of calendar time with three knots); and (ii) apparent, rather than observed, daily mean temperature (accounting for relative humidity and wind speed).[33]

Sex-disaggregated analysis was not appropriate for this study as all participants were of female sex. Gender-disaggregated analysis was not possible as gender information was not collected in the primary (PopDev) study.

For peer review only - http://bmjopen.bmj.com/site/about/guidelines.xhtml

Analyses were done in R 4.0.4,[34] using RStudio and the following R packages: lme4;[35] nlme;[36] stats;[34] splines;[34] effects;[37] ggplot2;[38] HeatStress;[33] Hmisc.[39]

Patient and public involvement

Stakeholders were involved during development of the original proposal in June 2012. The objectives and plans for the primary (PopDev) study were discussed with representatives from the community and reproductive health NGOs, as well as health professionals and policy makers at local and national levels, by means of a workshop, email, and telephone.

Stakeholders from the policy or associative arena presented their policies at the workshop to inform a group discussion on how best the study objectives could respond to their information needs. In another exercise, stakeholders were asked to identify one positive, one negative, and one surprising thing about the proposed study, which yielded particularly useful information when developing the proposal. We received feedback on substantive and methodological aspects of the project, and on communication issues, which was used to shape the study objectives and methodologies. One such change was the inclusion of the detailed daily activity questionnaire used in the secondary analysis herein.

The interview schedule was piloted with members of the community. Feedback on interview duration, meaningfulness and clarity of questions, and perceived gaps, was used to refine the wording of questions and to add/remove items. At the end of the primary (PopDev) study, a stakeholder consultation workshop took place to discuss the findings and their implications for cross-sectoral interventions, involving policy makers from different ministries and NGO staff.

Pregnant and postpartum women, as well as community members, in the Kaya and Bogodogo health districts of Burkina Faso were involved before the secondary study began. In-depth

For peer review only

For peer review only - http://bmjopen.bmj.com/site/about/guidelines.xhtml

interviews with pregnant and postpartum women (*n*=40), and focus group discussions with community members, were undertaken in October–November 2020.[7] The objectives for the secondary analysis were developed and informed by the lived experiences of postpartum women reported during this qualitative work. Specifically, women described how hot weather impedes breastfeeding due to excessive sweating and the discomfort of both mothers and their babies.[7]

Qualitative findings were discussed with stakeholders in maternal and neonatal health, climate change adaptation, as well as pregnant and postpartum women and community members, during a co-design workshop in Ouagadougou, Burkina Faso. Here, breastfeeding messaging was highlighted as an important area of focus for future research and interventions aimed at reducing the impact of heat stress on childbearing women and their newborn infants. We will continue our engagement with community members in the Kaya and Bogodogo health districts and will disseminate our findings through meetings, written summaries, and audio-visual materials. We will also engage with health decision-makers and provide summaries of the evidence and targeted policy briefs to support decision-makers in actions to reduce the impact of extreme high temperatures on maternal and neonatal health.

RESULTS

The population experienced year-round high temperatures, with an intra-annual range in daily mean temperature of 22.6–33.7°C. Figure S1 (supplementary material) shows daily minimum and maximum temperatures throughout the study period.

Eight-hundred and fifty-nine participants birthed 881 children (837 singleton births; 22 twin births). Six stillbirths, eight deaths in live born children, and 18 deaths in infants of unknown status at birth were reported. Seven women were lost to follow up between pregnancy and the postpartum period. The mean age of women at recruitment was 26.9 years (SD=6.2 years),

For peer review only - http://bmjopen.bmj.com/site/about/guidelines.xhtml

Table 1. Cohort characteristics, activities, and average daily temperature at each interview round.

	Interview round 1	Interview round 2	Interview round 3
Total interviewed (N)	839	810	792
Cohort characteristics			
*% urban [N] (NA)**	49.9 [419] (0)	49.3 [390] (19)	49.7 [389] (10)
% postpartum [N]	49.5 [415]	100 [810]	100 [792]
*% working in informal sector [N] (NA)**	37.6 [315] (1)	42.5 [343] (3)	48.9 [386] (2)
Living arrangements			
% with partner full-time [N]	80.9 [679]	80.1 [649]	80.1 [634]
% with partner periodically [N]	6.9 [58]	6.4 [52]	5.3 [42]
% not living with partner [N]	2.5 [21]	4.3 [35]	3.2 [25]
% not in a relationship [N]	0 [0]	8.8 [71]	7.7 [61]
% unknown [N]	9.7 [81]	0.4 [3]	3.8 [30]
Postpartum women only			
*% breastfeeding [N] (NA)**	100 [408] (7)	99.7 [782] (26)	99.7 [765] (25)
*% supplementary feeding [N] (NA)**		80.2 [628] (27)	98.0 [752] (25)
Time use (mins/day): *Breastfeeding (median [IQR]) (NA)**	120 [80–180] (11)	180 [120–180] (23)	240 [121–240] (26)
Time use (mins/day): *Childcare (median [IQR]) (NA)**	30 [15–40] (5)	30 [20–40] (11)	20 [15–30] (21)
Time use (mins/day): *Paid work/education (median [IQR])*	0 [0–0]	0 [0–92]	300 [0–420]
Time use (mins/day): *Domestic work (median [IQR])*	180 [110–240]	215 [145–300]	180 [130–235]
Infant age (weeks) (median [IQR]) *(NA)**	5.7 [2.6–9.9] (17)	12.6 [6.4–19.0] (25)	33.9 [27.1–41.0] (31)
Daily mean temperature (°C) (median [range]) *(NA)**	27.9 [22.7–32.8] (0)	27.0 [22.9–33.7] (11)	27.2 [23.3–30.3] (0)

Number (N) of women and % of total interviewed at each survey round, or summary statistics specified. * Missing values were excluded from calculations. NA = N missing. Where NA is not provided, N=0.

with a median gravidity of 3 pregnancies (IQR=2–5 pregnancies). Only 33 women (3.9%) were formally employed at baseline, of which 21 women were eligible for (or benefiting

For peer review only - http://bmjopen.bmj.com/site/about/guidelines.xhtml

from) maternity leave. Informal paid work was more common (see table 1). The most common occupations reported at baseline were trade/sales/small business (253 of 839 women), agriculture/farming (236 women), and housekeeping (218 women). Most women (808 of 839 interviewed at baseline) lived in houses with contemporary roof materials; primarily sheet metal (731 women) and timber (81 women).

The median time between first and second interviews was 92 days (IQR 80–108 days), and 149 days (IQR 141–155 days) between the second and third interviews. Total median follow-up time was 236 days (IQR 227–257 days). One woman refused to participate during the second round of interviews and one woman was travelling during the third round of interviews. The reasons why other women were lost to follow-up (n=6 at interview two, n=41 at interview three) are unknown.

The vast majority of postpartum women reported breastfeeding their infants at each interview round (table 1). However, the incidence of exclusive breastfeeding was low. Only 148 of 710 infants (20.8%) aged less than six months were exclusively breastfed on the day/night before interview two, and only 11 of 157 (7%) were exclusively breastfed before interview three.

On average, daily breastfeeding duration increased over time (figure 1a). After adjusting for long-term trends, a slight decrease in breastfeeding duration was observed as temperatures increased (figure 2a). Before adjusting for potential confounders (accounting only for the longitudinal and nested structure of the data), breastfeeding was estimated to decrease by 5.6 minutes/day (95% CI -7.0 to -4.1, p<0.001, n=783 women) per 1°C increase in same-day mean temperature. After controlling for important confounders (month of data collection, interview round, singleton/multiple birth, residential area, and time spent on paid work or education [minutes/day]), breastfeeding was estimated to decrease by 2.3 minutes/day (95% CI -4.6 to 0.04, p=0.05, n=783 women) per 1°C increase in same-day mean temperature

For peer review only - http://bmjopen.bmj.com/site/about/guidelines.xhtml

(table S2). This estimate was for infants aged 0.6–57 weeks (median=18.6 weeks). However, temperature interacted with infant age to affect breastfeeding duration (p=0.02). Time spent breastfeeding very young infants (four weeks) did not change with temperature. As infants aged, women were predicted to spend increasingly less time breastfeeding at high temperatures (figure 3).

On average, women spent less time on childcare at interview three (table 1), which coincided with the rainy and early dry/cooler seasons (figure 1b). After seasonal control, a slight increase in childcare time was observed as temperatures increased (figure 2b). Before adjustment, we estimated a 0.4-minute increase (0.1 to 0.8, p=0.02, n=814 women) in daily childcare per 1°C increase in mean temperature. We estimated a 0.6-minute increase (0.06 to 1.2, p=0.03, n=787 women) in childcare per 1°C increase in temperature after adjusting for month, interview round, singleton/multiple birth, infant age, maternal age, time spent on paid work or education (minutes/day]), and women's living arrangements (table S3).

A large proportion of women provided supplementary fluids to their infant (table 1); primarily water, herbal tea and, in the rainy season, boiled water. Milk other than breastmilk was rarely given. During interview round two (infant age 0–43 weeks), 30 of 43 women (70%) provided supplementary fluids on a relatively cool day, whereas 77 of 86 women (90%) provided supplementary fluids on a very hot day (figure 4a). Before adjusting for month or infant age, there was some indication of a positive relationship between temperature and supplementary feeding (OR=1.08, 0.99 to 1.18, p=0.08, n=632 women). After adjustment, the estimate was no longer statistically significant, although the direction of association remained the same (OR=1.06, 0.93 to 1.20, p=0.4, n=604 women). Infant age was the strongest predictor of supplementary feeding (OR=1.15, 1.11 to 1.19, p<0.001, n=604 women).

For peer review only - http://bmjopen.bmj.com/site/about/guidelines.xhtml

For women with infants aged less than six months at interview two, one third (12 of 36 women) reported exclusive breastfeeding on a ‘cool’ day (<26.5°C); whereas, only 12% (nine of 76 women) reported exclusive breastfeeding on a very hot day (>32.5°C) (figure 4b). Before confounder control, there was some evidence to suggest that women were less likely to exclusively breastfeed as temperature increased (OR=0.92, 0.84 to 1.01, *p*=0.07, *n*=576 women). After controlling for month and infant age, there was no strong evidence of association between daily mean temperature and exclusive breastfeeding (OR=0.93, 0.82 to 1.06, *p*=0.3, *n*=576 women).

There was no evidence of an interaction between temperature and residential area (urban/rural) or type of roofing materials on any outcome measured (*p*>0.05).

Estimated temperature effects were robust to sensitivity analyses. The main effect on breastfeeding duration was very robust and increased in statistical significance with alternative methods of seasonal control. The interaction between temperature and infant age on breastfeeding duration, and the main effect of temperature on daily childcare duration, were also fairly robust but with reduced significance.

DISCUSSION

This study explored the impacts of high ambient temperature on infant feeding practices among postpartum women in a low-income setting. We found a decrease in breastfeeding duration as temperatures increased; approximating to a 25-minute reduction in breastfeeding on the hottest, compared to coolest, days of the year. The extent of this impact largely depended on the age of the infant. From approximately four months onwards, we predicted an increasingly negative impact of high temperature on breastfeeding duration. For younger infants, temperature had a lower impact. There was no strong evidence that exclusive breastfeeding of young infants (aged less than six months) declines in very hot weather, or

For peer review only

For peer review only - http://bmjopen.bmj.com/site/about/guidelines.xhtml

that women are more likely to provide supplementary fluids to their infants (aged up to 12 months) as temperatures rise. However, women did tend to spend more time caring for their children at high temperatures.

There may be several explanations for the reduction in breastfeeding duration as temperatures rise in hot climates. Infants may demand less milk in order to limit heat-generation, or they may become too uncomfortable to feed. Mothers in the Kaya and Bogodogo health districts of Ouagadougou, Burkina Faso, report that their babies do not remain latched on during extreme heat due to the babies' discomfort.[7] On the other hand, mothers may offer less breastmilk due to their own discomfort under very hot conditions[7, 40] and/or due to a misperception that babies require supplementary water, especially on hot days.[8-10]

It is not unusual for breastfeeding patterns to change in hot weather. Infants may refuse to feed during the hottest part of the day and/or they may demand more frequent, but shorter, feeds throughout the day.[41] In doing so, babies consume mostly low-fat milk and avoid breast milk with a high fat content (i.e. afternoon/evening milk, last milk of a feed).[42] Ideally, the total intake of breast milk over a 24-hour period would increase during extreme hot weather in order to avoid infant dehydration. However, our findings tentatively suggest the contrary.

Many women across the world report perceived inadequacy of milk supply as their main reason for early weaning.[43] It is not clear if breast milk production is impacted by heat exposure, either directly or indirectly. High temperatures may exacerbate water stress, increasing the risk of dehydration among mothers in water-poor regions. However, the effects of maternal dehydration on breast milk production in hot climates are largely unknown.[44, 45] Several studies have shown that exclusively breastfed infants maintain normal hydration under hot conditions,[46-48] indicating that the quantity of breast milk is not affected.

For peer review only

For peer review only - http://bmjopen.bmj.com/site/about/guidelines.xhtml

However, field and experimental animal studies have shown that both the yield and nutritional composition of ruminant milk decline under hot conditions.[49] Further, the milk production capacity of animal mammary epithelial cells was found to decline following in vitro exposure to high temperatures (41°C).[50] To our knowledge, no studies have examined breast milk production in relation to temperature, but there is evidence that maternal stress affects breast milk composition[51] and delays secretory activation.[52] Even if milk supply is not adversely affected, high temperatures may contribute to women's perception of inadequate milk supply.

To our knowledge, this is the first study to quantify the acute effects of heat on breastfeeding, an important global health outcome. Studies in South America, South Asia, and Africa show seasonal differences in breastfeeding behaviour, with conflicting results.[22, 25-27] For example, in Bihar, India, infants under six months were more likely to be exclusively breastfed in the colder than warmer season.[26] Whereas, in rural Egypt, exclusive breastfeeding of infants aged 6–11 months was more prevalent in the hot than cool season.[22] However, such studies are not sufficient to demonstrate an effect of temperature as seasonal patterns are rarely driven by temperature alone, and changes in work availability and nutrition also need to be taken into account.

The main strengths of this study are the longitudinal dataset, population-based sampling, detailed questionnaire on activities, small loss to follow-up, and extensive confounder control. The main limitations are the small sample size, which may have reduced our ability to detect statistically significant associations (increasing the chances of a Type II error), and the short recruitment window (ideally, the study would have been conducted over several years). However, 2014 was a climatically typical year in Burkina Faso during 2010s.[21] Although our outcome measurements relied on self-reports, women were not asked directly if

For peer review only - http://bmjopen.bmj.com/site/about/guidelines.xhtml

they breastfed their infant exclusively. Instead, this outcome was constructed from women's recall of all fluids given to their child in the past 24 hours. Questions on infant feeding practices and childcare were embedded within an extensive interview schedule, further reducing the possibility of response bias. Nevertheless, our measurement of exclusive breastfeeding was not optimal. Finally, the outcome of childcare refers to time spent with children of all ages as this question was not specifically phrased to indicate the target (newborn) child.

Our findings are likely to be generalisable to Burkina Faso and other countries in the Sahel region, which share the same temperature conditions and have similar levels of breastfeeding uptake. Our findings may also be generalisable to other regions of Africa with a similar climate and breastfeeding uptake if, as proposed, breastfeeding duration is reduced at high temperatures due to infant or maternal discomfort and/or due to a perception that babies require water in hot weather. Mothers in West and East African countries (Burkina Faso and Kenya, respectively) report similar challenges when breastfeeding in extreme heat,[7,40] and hot weather has been identified as a barrier to optimal infant feeding practices in the Democratic Republic of the Congo (Central Africa)[8] and Ghana (West Africa).[9]

Larger studies are needed to further examine the impacts of heat on infant feeding practices in hot climates. Future research should consider temperature in relation to the number and duration of individual breastfeeds, and to the volume of breast milk and supplementary fluids consumed by infants, over a 24-hour period. Research should also seek to determine if high temperatures impact on breast milk production. Actions should be taken to ensure that hot weather does not negatively impact on breastfeeding behaviour. Effective interventions are likely to require a multidimensional approach.[53] It is important that health workers and

For peer review only - http://bmjopen.bmj.com/site/about/guidelines.xhtml

mothers are informed about normal heat-induced changes in infant breastfeeding patterns so that such changes are not misinterpreted as a need for supplementation.

CONCLUSIONS

Exclusive breastfeeding is an essential cornerstone for the wellbeing and survival of infants. Our findings suggest a substantial decrease in breastfeeding duration during hot weather. This finding is important as infants require increased hydration to cope physiologically with increased heat, and the safest form of hydration for young infants is breast milk.

Larger studies are needed in Burkina Faso and beyond as climate change in Africa is accelerating.[54] Without effective interventions, mothers may find it increasingly difficult to breastfeed their infants as temperatures rise. Maternal and child health programmes in hot climates should be updated to improve messaging and breastfeeding practices during extreme hot weather.

For peer review only

406 **Acknowledgements**

407 We thank Maurice Yaogo, Patrick Ilbouldo, André Soubeiga, Katerini Storeng, Denis
408 Ouedraogo, Seydou Drabo, and Tim Powell-Jackson for their valuable contribution to the
409 primary (PopDev) study. We also gratefully acknowledge all women who participated in the
410 PopDev study.

411 **Contributors**

412 VF and SK conceived the study (secondary analysis). JAC and RG collected data. CP
413 conducted the statistical analysis under the supervision of SH. All authors interpreted the
414 results. CP drafted the paper. All authors critically revised the paper for important intellectual
415 content and approved the final version for publication. All authors agreed to be accountable
416 for all aspects of the work.

417 **Funding**

418 The PopDev study was supported by the Economic and Social Research Council (ESRC) in
419 response to the Joint ESRC-WOTRO-RCN-PRB-Hewlett call on Population & Development
420 [grant number ES/K011049/1]. The secondary analysis was supported by the Natural
421 Environment Research Council (NERC) [grant numbers NE/T013613/1, NE/T01363X/1];
422 Research Council of Norway (RCN) [grant number 312601]; and The Swedish Research
423 Council for Health, Working Life and Welfare in collaboration with the Swedish Research
424 Council (Forte) [grant number 2019-01570]; coordinated through a Belmont Forum
425 partnership.

426 **Competing interests**

427 None declared.

For peer review only

Ethics approval

The study was approved by the Research Ethics Committees of Centre Muraz, Burkina Faso (reference: A16-2013/CE-CM) and the London School of Hygiene & Tropical Medicine, United Kingdom (reference: 6401). The free and informed consent of each participant was obtained before each interview.

Data sharing statement

Individual-level deidentified participant data from the primary (PopDev) study are available to researchers who have a valid research question, which is not being investigated by the primary research team. A data sharing agreement will be required. All data requests should be made via https://datacompass.lshtm.ac.uk/id/eprint/64/. The questionnaires, consent form, data dictionary (codebook), and user guide are publicly available from https://datacompass.lshtm.ac.uk/id/eprint/64/. Data have been available from 2018 with no end date. Meteorological data are publicly available from TuTiempo (https://www.tutiempo.net/). Statistical code is available from the corresponding author.

For peer review only

REFERENCES

1 Opoku SK, Filho WL, Hubert F, et al. Climate change and health preparedness in Africa: Analysing trends in six African countries. *Int J Environ Res Public Health* 2021;18:4672 doi: 10.3390/ijerph18094672.

2 Baker RE, Anttila-Hughes J. Characterizing the contribution of high temperatures to child undernourishment in Sub-Saharan Africa. *Sci Rep* 2020;10:18796–96 doi: 10.1038/s41598-020-74942-9.

3 Sambou M-JG, Pohl B, Janicot S, et al. Heat waves in spring from Senegal to Sahel: Evolution under climate change. *Int J Climatol* 2021;41:6238–53 doi: 10.1002/joc.7176.

4 Roos N, Kovats S, Hajat S, et al. Maternal and newborn health risks of climate change: A call for awareness and global action. *Acta Obstet Gynecol Scand* 2021;100:566–70 doi: 10.1111/aogs.14124.

5 Chersich MF, Pham MD, Areal A, et al. Associations between high temperatures in pregnancy and risk of preterm birth, low birth weight, and stillbirths: systematic review and meta-analysis. *BMJ* 2020;371:m3811 doi: 10.1136/bmj.m3811.

6 Romero González AM, Belemvire A, Saulière S. Climate Change and Women Farmers in Burkina Faso: Impact and adaptation policies and practices. Oxfam Research ReportOxford, UK: Oxfam; 2011.

7 Kadio K, Mariam C, Scorgie F, et al. The experience of heat stress among pregnant and postpartum women and their neonates in Burkina Faso: a qualitative analysis. *Trop Med Int Health* 2021;26(Suppl 1):253–54 doi: 10.1111/tmi.13633.

8 Burns J, Emerson JA, Amundson K, et al. A qualitative analysis of barriers and facilitators to optimal breastfeeding and complementary feeding practices in South Kivu, Democratic Republic of Congo. *Food Nutr Bull* 2016;37:119–31 doi: 10.1177/0379572116637947.

For peer review only - http://bmjopen.bmj.com/site/about/guidelines.xhtml

9 Nsiah-Asamoah C, Doku DT, Agblorti S. Mothers' and Grandmothers' misconceptions and socio-cultural factors as barriers to exclusive breastfeeding: A qualitative study involving Health Workers in two rural districts of Ghana. *PLOS ONE* 2020;15:e0239278 doi: 10.1371/journal.pone.0239278.

10 Mundagowa PT, Chadambuka EM, Chimberengwa PT, et al. Barriers and facilitators to exclusive breastfeeding practices in a disadvantaged community in southern Zimbabwe: A maternal perspective. *World Nutrition* 2021;12:73–91 doi: 10.26596/wn.202112173-91.

11 Walters DD, Phan LTH, Mathisen R. The cost of not breastfeeding: global results from a new tool. *Health Policy Plan* 2019;34:407–17 doi: 10.1093/heapol/czz050.

12 Wells JC, Sawaya AL, Wibaek R, et al. The double burden of malnutrition: aetiological pathways and consequences for health. *Lancet* 2020;395:75–88 doi: 10.1016/S0140-6736(19)32472-9.

13 Victora CG, Bahl R, Barros AJD, et al. Breastfeeding in the 21st century: epidemiology, mechanisms, and lifelong effect. *Lancet* 2016;387:475–90 doi: 10.1016/S0140-6736(15)01024-7.

14 World Health Organization. Infant and Young Child Feeding. 2021. https://www.who.int/en/news-room/fact-sheets/detail/infant-and-young-child-feeding (accessed 10 August 2021).

15 Issaka AI, Agho KE, Renzaho AM. Prevalence of key breastfeeding indicators in 29 sub-Saharan African countries: a meta-analysis of demographic and health surveys (2010–2015). *BMJ Open* 2017;7:e014145 doi: 10.1136/bmjopen-2016-014145.

16 Nankumbi J, Muliira JK. Barriers to infant and child-feeding practices: a qualitative study of primary caregivers in Rural Uganda. *J Health Popul Nutr* 2015;33:106–16.

For peer review only - http://bmjopen.bmj.com/site/about/guidelines.xhtml

17 Matare C, Craig H, Martin S, et al. Barriers and opportunities for improved exclusive breast-feeding practices in Tanzania: Household trials with mothers and fathers. *Food Nutr Bull* 2019;40:308-25 doi: 10.1177/0379572119841961.

18 Moyo G, Magaisa T, Pagiwa A, et al. Barriers and facilitators of exclusive breastfeeding: Findings from a barrier analysis conducted in Mwenezi and Chiredzi Districts, Zimbabwe. *World Nutrition* 2020;11:12–21 doi: 10.26596/wn.202011312-21.

19 Randell H, Grace K, Bakhtsiyarava M. Climatic conditions and infant care: implications for child nutrition in rural Ethiopia. *Popul Environ* 2021;42:524–52 doi: 10.1007/s11111-020-00373-3.

20 Weber M. Burkina Faso Jobs Diagnostic: Overview and Suggestions for a Strategic Framework for Jobs. Jobs Series;15. Washington DC, USA: World Bank Group; 2018. https://openknowledge.worldbank.org/handle/10986/31033 (accessed 15 December 2021).

21 World Bank Group. Climate Change Knowledge Portal. Burkina Faso. Current Climate. 2021. https://climateknowledgeportal.worldbank.org/country/burkina-faso/climate-data-historical (accessed 20 Oct 2021).

22 Serdula MK, Seward J, Marks JS, et al. Seasonal differences in breast-feeding in rural Egypt. *Am J Clin Nutr* 1986;44:405–09 doi: 10.1093/ajcn/44.3.405.

23 Simondon KB, F. S. Mothers prolong breastfeeding of undernourished children in rural Senegal. *Int J Epidemiol* 1998;27:490-4 doi: 10.1093/ije/27.3.490.

24 Dede KS, Bras H. Exclusive breastfeeding patterns in Tanzania: Do individual, household, or community factors matter? *Int Breastfeed J* 2020;15:32 doi: 10.1186/s13006-020-00279-8.

25 González-Chica DA, Gonçalves H, Nazmi A, et al. Seasonality of infant feeding practices in three Brazilian birth cohorts. *Int J Epidemiol* 2012;41:743–52 doi: 10.1093/ije/dys002.

For peer review only

26 Das A, Chatterjee R, Karthick M, et al. The influence of seasonality and community-based health worker provided counselling on exclusive breastfeeding - findings from a cross-sectional survey in India. *PLOS ONE* 2016;11:e0161186 doi: 10.1371/journal.pone.0161186.

27 Hossain MM, Radwan MM, Arafa SA, et al. Prelacteal Infant Feeding Practices in Rural Egypt. *J Trop Pediatr* 1992;38:317–22 doi: 10.1093/tropej/38.6.317.

28 Fitzpatrick RGJ, Parker DJ, Marsham JH, et al. How a typical West African day in the future-climate compares with current-climate conditions in a convection-permitting and parameterised convection climate model. *Clim Change* 2020;163:267–96 doi: 10.1007/s10584-020-02881-5.

[dataset] [29] Cresswell J, Drabo S, Filippi V, et al. Data from: Productivity, family planning and reproductive health in Burkina Faso: the PopDev study. LSHTM Data Compass, 2018. https://doi.org/10.17037/DATA.13.

30 Institut National de la Statistique et de la Démographie (INSD). Cinquième Recensement Général de la Population et de l'Habitation du Burkina Faso: Résultats Préliminaires. 2020. https://www.insd.bf/contenu/documents_rgph5/RAPPORT_PRELIMINAIRE_RGPH_2019.pdf (accessed 15 August 2021).

31 Beck HE, Zimmermann NE, McVicar TR, et al. Present and future Köppen-Geiger climate classification maps at 1-km resolution. *Sci Data* 2018;5:180214 doi: 10.1038/sdata.2018.214.

[dataset] [32] TuTiempo. Data from: Clima Bobo-Dioulasso. Datos climáticos: 1973 - 2021. https://www.tutiempo.net/clima/ws-655100.html.

33 Ana, MeteoSwiss. anacv/HeatStress: zenodo (v1.0.7_zenodo) [program]. Zenodo, 2019. doi: 10.5281/zenodo.3264929.

34 R Core Team. R: A language and environment for statistical computing [program]. R Foundation for Statistical Computing, 2021. https://www.R-project.org/.

For peer review only

For peer review only - http://bmjopen.bmj.com/site/about/guidelines.xhtml

35 Bates D, Maechler M, Bolker B, et al. Fitting linear mixed-effects models using lme4. *J Stat Softw* 2015;67:1–48 doi: 10.18637/jss.v067.i01.

36 Pinheiro J, Bates D, DebRoy S, et al. nlme: Linear and Nonlinear Mixed Effects Models. R package version 3.1-152 [program]. CRAN, 2021. https://CRAN.R-project.org/package=nlme.

37 Fox J. Effect displays in R for generalised linear models. *J Stat Softw* 2003;8:1–27.

38 Wickham H. ggplot2: Elegant Graphics for Data Analysis. New York: Springer-Verlag 2016.

39 Harrell FE Jr. Hmisc: Harrell Miscellaneous. R package version 4.6-0. [program]. CRAN, 2021. https://CRAN.R-project.org/package=Hmisc.

40 Lusambili A, Khaemba P, Chabeda S, et al. Community perspectives on maternal and newborn experiences of heat stress: a qualitative inquiry in Kilifi, Kenya. *Trop Med Int Health* 2021;26(Suppl 1):253. doi: 10.1111/tmi.13633.

41 Australian Breastfeeding Association. Breastfeeding through warmer weather. 2017. https://www.breastfeeding.asn.au/bf-info/you-and-your-breastfed-baby/cool (accessed 08 Nov 2021).

42 Ballard O, Morrow AL. Human milk composition: nutrients and bioactive factors. *Pediatr Clin North Am* 2013;60:49–74 doi: 10.1016/j.pcl.2012.10.002.

43 Gatti L. Maternal perceptions of insufficient milk supply in breastfeeding. *J Nurs Scholarsh* 2008;40:355–63 doi: 10.1111/j.1547-5069.2008.00234.x.

44 Bentley GR. Hydration as a limiting factor in lactation. *Am J Hum Biol* 1998;10:151–61 doi: 10.1002/(SICI)1520-6300(1998)10:2<151::AID-AJHB2>3.0.CO;2-O.

45 Schuster RC, Butler MS, Wutich A, et al. “If there is no water, we cannot feed our children”: The far-reaching consequences of water insecurity on infant feeding practices and

For peer review only

For peer review only - http://bmjopen.bmj.com/site/about/guidelines.xhtml

infant health across 16 low- and middle-income countries. *Am J Hum Biol* 2020;32:e23357 doi: 10.1002/ajhb.23357.

46 Sachdev HP, Krishna J, Puri RK. Do exclusively breast fed infants need fluid supplementation? *Indian Pediatr* 1992;29:535-40.

47 Ashraf RN, Jalil F, Aperia A, et al. Additional water is not needed for healthy breast-fed babies in a hot climate. *Acta Paediatr* 1993;82:1007–11 doi: 10.1111/j.1651-2227.1993.tb12799.x.

48 Cohen RJ, Brown KH, Rivera LL, et al. Exclusively breastfed, low birthweight term infants do not need supplemental water. *Acta Paediatr* 2000;89:550-52 doi: 10.1080/080352500750027835.

49 Bernabucci U, Basiricò L, Morera P. Impact of hot environment on colostrum and milk composition. *Cell Mol Biol* 2013;59:67–83 doi: 10.1170/T948.

50 Kobayashi K, Tsugami Y, Matsunaga K, et al. Moderate high temperature condition induces the lactation capacity of mammary epithelial cells through control of STAT3 and STAT5 signaling. *J Mammary Gland Biol Neoplasia* 2018;23:75–88 doi: 10.1007/s10911-018-9393-3.

51 Ziomkiewicz A, Babiszewska M, Apanasewicz A, et al. Psychosocial stress and cortisol stress reactivity predict breast milk composition. *Sci Rep* 2021;11:11576 doi: 10.1038/s41598-021-90980-3.

52 Caparros-Gonzalez RA, Romero-Gonzalez B, Gonzalez-Perez R, et al. Maternal and neonatal hair cortisol levels and psychological stress are associated with onset of secretory activation of human milk production. *Adv Neonatal Care* 2019;19:E11–20 doi: 10.1097/ANC.0000000000000660.

For peer review only

For peer review only - http://bmjopen.bmj.com/site/about/guidelines.xhtml

584 53 Cresswell JA, Ganaba R, Sarrassat S, et al. The effect of the Alive & Thrive initiative on exclusive breastfeeding in rural Burkina Faso: a repeated cross-sectional cluster randomised controlled trial. *Lancet Glob Health* 2019;7:e357–65 doi: 10.1016/S2214-109X(18)30494-7.

587 54 IPCC. Summary for Policymakers. In: MassonDelmotte V, Zhai P, Pirani A, et al., eds. Climate Change 2021: The Physical Science Basis Contribution of Working Group I to the Sixth Assessment Report of the Intergovernmental Panel on Climate Change. Cambridge, UK: Cambridge University Press, In press.

For peer review only

For peer review only - http://bmjopen.bmj.com/site/about/guidelines.xhtml

Figure legends

Figure 1. Average time spent (minutes per day) (a) breastfeeding and (b) caring for children over time, with fitted natural cubic splines of time (dashed lines). Blue shading = dry, cooler season (November–February), red shading = dry, hot season (March–May), green shading = rainy season (June–October). Data source: PopDev study.[29]

Figure 2. Scatterplots of daily mean temperature (°C) and standardised residuals from fitted trends (natural cubic splines of time) in the (a) breastfeeding and (b) childcare time-series, with locally weighted smoothing (blue line) and 95% confidence intervals (grey shading).

Figure 3. Effects of daily mean temperature (°C) on time spent breastfeeding (minutes per day) at specified infant ages (4, 13, 26, 52 weeks), as predicted from an autoregressive multilevel linear model with confounder control and an interaction term between temperature and infant age.

Figure 4. Incidence of (a) supplementary feeding and (b) exclusive breastfeeding in past 24h among women interviewed following the hottest and coolest days of the season. Blue = days below the 10th percentile of daily mean temperature between March–June 2014. Red = days above the 90th percentile of daily mean temperature between March–June 2014.

For peer review only - http://bmjopen.bmj.com/site/about/guidelines.xhtml

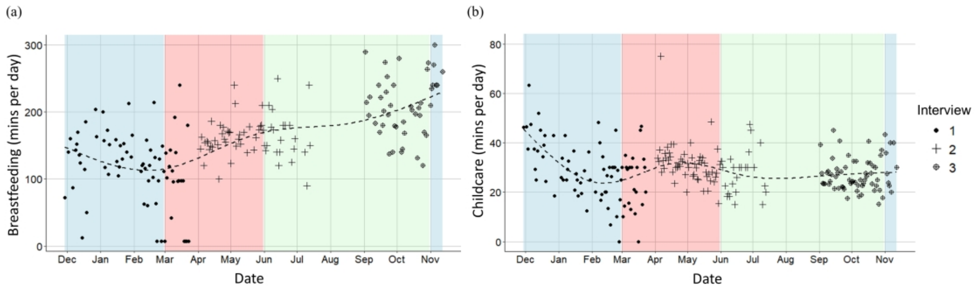

Figure 1. Average time spent (minutes per day) (a) breastfeeding and (b) caring for children over time, with fitted natural cubic splines of time (dashed lines). Blue shading = dry, cooler season (November–February), red shading = dry, hot season (March–May), green shading = rainy season (June–October). Data source: PopDev study.[29]

338x190mm (300 x 300 DPI)

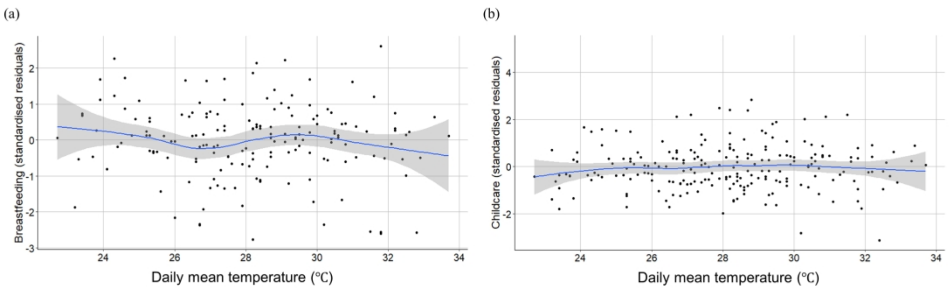

Figure 2. Scatterplots of daily mean temperature (°C) and standardised residuals from fitted trends (natural cubic splines of time) in the (a) breastfeeding and (b) childcare time-series, with locally weighted smoothing (blue line) and 95% confidence intervals (grey shading).

338x190mm (300 x 300 DPI)

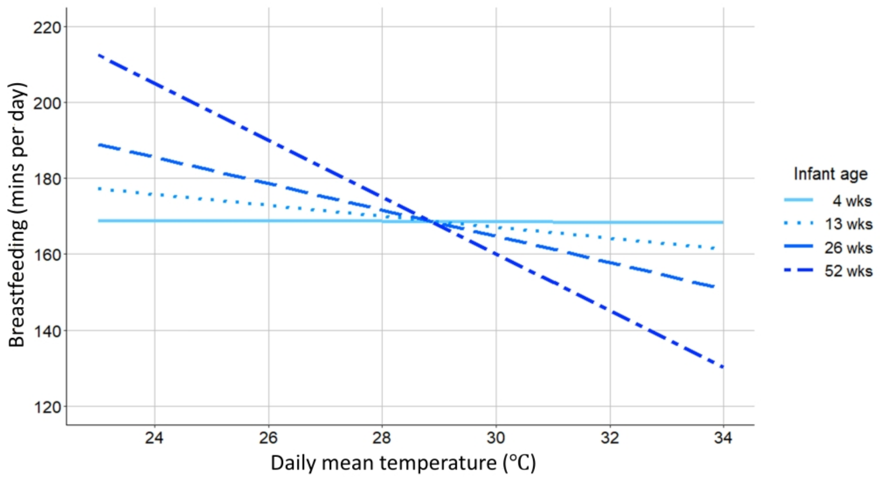

Figure 3. Effects of daily mean temperature (°C) on time spent breastfeeding (minutes per day) at specified infant ages (4, 13, 26, 52 weeks), as predicted from an autoregressive multilevel linear model with confounder control and an interaction term between temperature and infant age.

338x190mm (300 x 300 DPI)

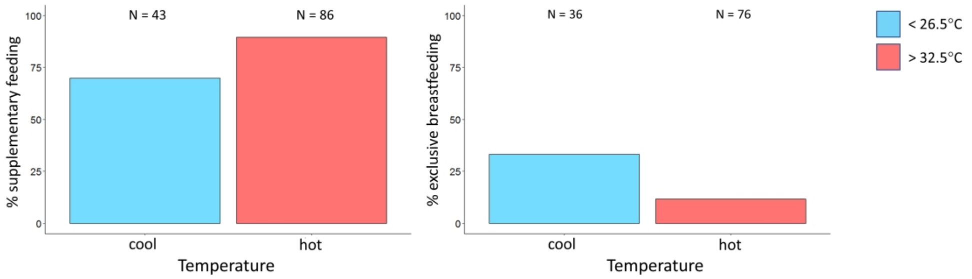

Figure 4. Incidence of (a) supplementary feeding and (b) exclusive breastfeeding in past 24h among women interviewed following the hottest and coolest days of the season. Blue = days below the 10th percentile of daily mean temperature between March–June 2014. Red = days above the 90th percentile of daily mean temperature between March–June 2014.

338x190mm (300 x 300 DPI)

SUPPLEMENTAL MATERIAL

How do high ambient temperatures affect infant feeding practices? A prospective cohort study of postpartum women in Bobo-Dioulasso, Burkina Faso

Contents

Table S1. Outcomes, exposure, potential confounders and covariates considered in all models. 2

Figure S1. Daily maximum and minimum temperatures and seasons in Bobo-Dioulasso, Burkina Faso 3

Table S2. Effect estimates, 95% confidence intervals, and *p*-values of each variable included in the multilevel linear model of temperature-breastfeeding association 4

Table S3. Effect estimates, 95% confidence intervals, and *p*-values of each variable included in the multilevel linear model of temperature-childcare association 5

References 5

For peer review only

For peer review only - http://bmjopen.bmj.com/site/about/guidelines.xhtml

Table S1. Outcomes, exposure, potential confounders and covariates considered in all models.

Variable	Definition	Units	Type	Model
**Outcomes**				
Breastfeeding duration	Time spent breastfeeding infants aged 4+ days	Minutes per day	Continuous	MLM (BF)
Exclusive breastfeeding	No liquids other than breast milk given in past 24h (infants aged <6 months)	Yes, No	Binary	Logistic (EBF)
Supplementary feeding	Any liquid other than breast milk given in past 24h (infants aged <12 months)	Yes, No	Binary	Logistic (SF)
Childcare duration	Time caring for (clothing, etc) children aged 0+ days	Minutes per day	Continuous	MLM (CH)
**Exposure**				
Daily mean temperature	Mean temperature in Bobo-Dioulasso on day before interview	°C	Continuous	All
**Confounders**				
Season/time trends	Seasonal patterns (unrelated to temperature) and long-term trends	Month of interview	Categorical	All
Interview round	Interview (baseline, 3-months post-baseline, 9-months post-baseline)	1, 2, 3	Categorical	BF, CH
Infant age	Date of interview – Date of delivery	Weeks	Continuous	All
Income-generating work	Time spent on professional or educational activities	Minutes per day	Continuous	All
Domestic work	Time spent on domestic activities (excluding family care)	Minutes per day	Continuous	BF, EBF, SF
**Covariates**				
Number born	Singleton or multiple birth	1, 2	Categorical	All
Gravidity	Total number of pregnancies, including current	1, 2–5, 6+ pregnancies	Categorical	All
Maternal age	Age of mother at baseline	≤ 19, 20–34, ≥ 35 years	Categorical	All
Living arrangements	Mothers' living arrangements with partner at each interview	With partner full-time, With partner periodically, Not with partner, Not in a relationship	Categorical	CH
Residential area	Mother's area of residence	Urban, Rural	Categorical	All
Roofing materials	Roofing materials of mother's house	Natural, Rudimentary, Contemporary	Categorical	All
**Interactions**				
*Temperature * Residential area*	Daily mean temperature and mother's area of residence	°C * Urban/Rural	Interaction	All
*Temperature * Infant age*	Daily mean temperature and infant age	°C * Weeks	Interaction	All
*Temperature * Roofing materials*	Daily mean temperature and roofing materials of mother's house	°C * Natural/Rudimentary/ Contemporary	Interaction	All

MLM = Multilevel model. BF = Breastfeeding duration. CH = Childcare duration. SF = Supplementary feeding. EBF = Exclusive breastfeeding.

For peer review only

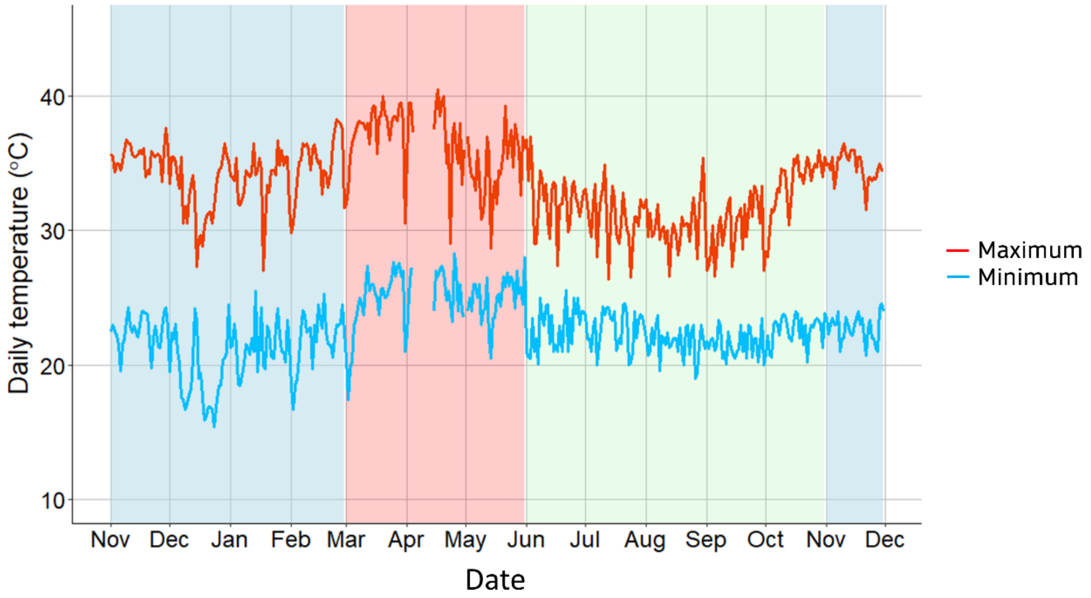

Figure S1. Daily maximum and minimum temperatures and seasons in Bobo-Dioulasso, Burkina Faso, between 1st Nov 2013 and 30th Nov 2014. Blue = dry, cooler season (November–February), red = dry, hot season (March–May), green = rainy season (June–October). Data source: TuTiempo.net [1].

For peer review only

Table S2. Effect estimates, 95% confidence intervals, and *p*-values of each variable included in the autoregressive multilevel linear model for the exposure-response association between temperature and breastfeeding duration (minutes/day).

Variable	Estimate	95% CI		*p*-value
Daily mean temperature (°C)	-2.29	-4.63	0.04	0.05
Interview round (reference: 1)				
2	32.01	-52.53	116.54	0.46
3	135.46	59.35	211.57	<0.001
Month of data collection (reference: January)				
February	-34.16	-61.25	-7.07	0.01
March	-61.79	-89.14	-34.45	<0.001
April	-7.70	-95.20	79.79	0.86
May	-22.75	-106.68	61.18	0.59
June	-37.01	-122.13	48.12	0.39
July	-37.57	-135.16	60.02	0.45
September	-89.72	-167.34	-12.09	0.02
October	-102.25	-178.37	-26.14	<0.01
November	-66.34	-136.35	3.66	0.06
December	-13.72	-35.25	7.81	0.21
Singleton/multiple birth (reference: singleton)				
Multiple birth	70.46	48.43	92.49	< 0.001
Residential area (reference: rural)				
Urban	18.26	-5.76	42.29	0.13
Income-generating activities (minutes/day)	0.02	0.00	0.04	0.02

For peer review only

Table S3. Effect estimates, 95% confidence intervals, and *p*-values of each variable included in the autoregressive multilevel linear model for the exposure-response association between temperature and childcare duration (minutes/day).

Variable	Estimate	95% CI		*p*-value
Daily mean temperature (°C)	0.65	0.06	1.24	0.03
Interview round (reference: 1)				
2	-8.09	-34.64	18.47	0.55
3	-15.25	-36.13	5.63	0.15
Month of data collection (reference: January)				
February	-1.75	-7.64	4.13	0.56
March	-4.36	-10.33	1.60	0.15
April	9.96	-17.18	37.09	0.47
May	8.99	-17.90	35.87	0.51
June	8.32	-18.84	35.48	0.55
July	3.48	-25.75	32.71	0.82
September	15.08	-5.95	36.10	0.16
October	12.88	-8.13	33.90	0.23
November	16.52	-2.63	35.66	0.09
December	14.34	8.75	19.92	< 0.001
Singleton/multiple birth (reference: singleton)				
Multiple birth	17.43	11.53	23.33	< 0.001
Infant age (weeks)	0.09	-0.04	0.21	0.17
Maternal age (reference: 20-34 years)				
< 19 years	-5.68	-8.68	-2.69	< 0.001
≥ 35 years	0.29	-2.39	2.97	0.83
Income-generating activities (minutes/day)	-0.01	-0.01	-0.00	0.04
Living arrangements (reference: Lives with partner full-time)				
Lives with partner periodically	-1.91	-5.73	1.92	0.33
Does not live with partner	-2.57	-7.42	2.29	0.30
Not in a relationship	-8.68	-12.47	-4.89	< 0.001

References

1 TuTiempo. Clima Bobo-Dioulasso. Datos climáticos: 1973 - 2021. https://www.tutiempo.net/clima/ws-655100.html.

For peer review only - http://bmjopen.bmj.com/site/about/guidelines.xhtml

STROBE Statement—Checklist of items that should be included in reports of ***cohort studies***

	Item No	Recommendation	Page No
**Title and abstract**	1	(*a*) Indicate the study's design with a commonly used term in the title or the abstract	1
		(*b*) Provide in the abstract an informative and balanced summary of what was done and what was found	3-4
**Introduction**			
Background/rationale	2	Explain the scientific background and rationale for the investigation being reported	2-4
Objectives	3	State specific objectives, including any prespecified hypotheses	7
**Methods**			
Study design	4	Present key elements of study design early in the paper	7
Setting	5	Describe the setting, locations, and relevant dates, including periods of recruitment, exposure, follow-up, and data collection	8, 9
Participants	6	(*a*) Give the eligibility criteria, and the sources and methods of selection of participants. Describe methods of follow-up	8, 9
		(*b*) For matched studies, give matching criteria and number of exposed and unexposed	N/A
Variables	7	Clearly define all outcomes, exposures, predictors, potential confounders, and effect modifiers. Give diagnostic criteria, if applicable	Table S1
Data sources/ measurement	8*	For each variable of interest, give sources of data and details of methods of assessment (measurement). Describe comparability of assessment methods if there is more than one group	8-9
Bias	9	Describe any efforts to address potential sources of bias	8-9, 11
Study size	10	Explain how the study size was arrived at	N/A
Quantitative variables	11	Explain how quantitative variables were handled in the analyses. If applicable, describe which groupings were chosen and why	10-11
Statistical methods	12	(*a*) Describe all statistical methods, including those used to control for confounding	10-11
		(*b*) Describe any methods used to examine subgroups and interactions	11
		(*c*) Explain how missing data were addressed	9, 11
		(*d*) If applicable, explain how loss to follow-up was addressed	11
		(*e*) Describe any sensitivity analyses	11
**Results**			
Participants	13*	(a) Report numbers of individuals at each stage of study—eg numbers potentially eligible, examined for eligibility, confirmed eligible, included in the study, completing follow-up, and analysed	13, Table 1, 15, 16, 17
		(b) Give reasons for non-participation at each stage	15
		(c) Consider use of a flow diagram	
Descriptive data	14*	(a) Give characteristics of study participants (eg demographic, clinical, social) and information on exposures and potential confounders	Table 1, 13-15
		(b) Indicate number of participants with missing data for each variable of interest	Table 1
		(c) Summarise follow-up time (eg, average and total amount)	15
Outcome data	15*	Report numbers of outcome events or summary measures over time	Table 1, 15

For peer review only - http://bmjopen.bmj.com/site/about/guidelines.xhtml

Main results	16	(*a*) Give unadjusted estimates and, if applicable, confounder-adjusted estimates and their precision (eg, 95% confidence interval). Make clear which confounders were adjusted for and why they were included	15-17, 11, Table S1
		(*b*) Report category boundaries when continuous variables were categorized	N/A
		(*c*) If relevant, consider translating estimates of relative risk into absolute risk for a meaningful time period	N/A
Other analyses	17	Report other analyses done—eg analyses of subgroups and interactions, and sensitivity analyses	16, 17
**Discussion**			
Key results	18	Summarise key results with reference to study objectives	17-18
Limitations	19	Discuss limitations of the study, taking into account sources of potential bias or imprecision. Discuss both direction and magnitude of any potential bias	19-20
Interpretation	20	Give a cautious overall interpretation of results considering objectives, limitations, multiplicity of analyses, results from similar studies, and other relevant evidence	18-19
Generalisability	21	Discuss the generalisability (external validity) of the study results	20
**Other information**			
Funding	22	Give the source of funding and the role of the funders for the present study and, if applicable, for the original study on which the present article is based	22

*Give information separately for exposed and unexposed groups.

Note: An Explanation and Elaboration article discusses each checklist item and gives methodological background and published examples of transparent reporting. The STROBE checklist is best used in conjunction with this article (freely available on the Web sites of PLoS Medicine at http://www.plosmedicine.org/, Annals of Internal Medicine at http://www.annals.org/, and Epidemiology at http://www.epidem.com/). Information on the STROBE Initiative is available at http://www.strobe-statement.org.

For peer review only

For peer review only - http://bmjopen.bmj.com/site/about/guidelines.xhtml

BMJ Open

How do high ambient temperatures affect infant feeding practices? A prospective cohort study of postpartum women in Bobo-Dioulasso, Burkina Faso

Journal:	*BMJ Open*
Manuscript ID	bmjopen-2022-061297.R1
Article Type:	Original research
Date Submitted by the Author:	11-Aug-2022
Complete List of Authors:	Part, Chérie; London School of Hygiene & Tropical Medicine, Department of Public Health, Environments and Society<br>Filippi, Veronique; London School of Hygiene & Tropical Medicine, Department of Infectious Disease Epidemiology<br>Cresswell, Jenny; London School of Hygiene & Tropical Medicine, Department of Infectious Disease Epidemiology<br>Ganaba, Rasmané; AFRICSanté<br>Hajat, Shakoor; London School of Hygiene & Tropical Medicine, Department of Public Health, Environments and Society<br>Nakstad, Britt; University of Oslo, Institute of Clinical Medicine; University of Botswana, Department of Pediatrics and Adolescent Health<br>Roos, Nathalie; Karolinska Institutet, Department of Medicine<br>Kadio, Kadidiatou; Institut de Recherche en Sciences de la Santé<br>Chersich, Matthew; University of the Witwatersrand, Wits Reproductive Health and HIV Institute<br>Lusambili, Adelaide; Aga Khan University - Kenya, Department of Population Health<br>Kouanda, Seni; Institut de Recherche en Sciences de la Sante<br>Kovats, Sari; London School of Hygiene & Tropical Medicine, Department of Public Health, Environments and Society
**Primary Subject Heading**:	Public health
Secondary Subject Heading:	Epidemiology
Keywords:	EPIDEMIOLOGY, NUTRITION & DIETETICS, Community child health < PAEDIATRICS, PUBLIC HEALTH

SCHOLARONE™ Manuscripts

I, the Submitting Author has the right to grant and does grant on behalf of all authors of the Work (as defined in the below author licence), an exclusive licence and/or a non-exclusive licence for contributions from authors who are: i) UK Crown employees; ii) where BMJ has agreed a CC-BY licence shall apply, and/or iii) in accordance with the terms applicable for US Federal Government officers or employees acting as part of their official duties; on a worldwide, perpetual, irrevocable, royalty-free basis to BMJ Publishing Group Ltd ("BMJ") its licensees and where the relevant Journal is co-owned by BMJ to the co-owners of the Journal, to publish the Work in this journal and any other BMJ products and to exploit all rights, as set out in our licence.

The Submitting Author accepts and understands that any supply made under these terms is made by BMJ to the Submitting Author unless you are acting as an employee on behalf of your employer or a postgraduate student of an affiliated institution which is paying any applicable article publishing charge ("APC") for Open Access articles. Where the Submitting Author wishes to make the Work available on an Open Access basis (and intends to pay the relevant APC), the terms of reuse of such Open Access shall be governed by a Creative Commons licence – details of these licences and which Creative Commons licence will apply to this Work are set out in our licence referred to above.

Other than as permitted in any relevant BMJ Author's Self Archiving Policies, I confirm this Work has not been accepted for publication elsewhere, is not being considered for publication elsewhere and does not duplicate material already published. I confirm all authors consent to publication of this Work and authorise the granting of this licence.

For peer review only

HOW DO HIGH AMBIENT TEMPERATURES AFFECT INFANT FEEDING PRACTICES? A PROSPECTIVE COHORT STUDY OF POSTPARTUM WOMEN IN BOBO-DIOULASSO, BURKINA FASO

Chérie Part*[1], Véronique Filippi[2], Jenny A. Cresswell[2], Rasmané Ganaba[3], Shakoor Hajat[1], Britt Nakstad[4,5], Nathalie Roos[6], Kadidiatou Kadio[7], Matthew Chersich[8], Adelaide Lusambili[9], Seni Kouanda[7], Sari Kovats[1]

[1] Department of Public Health, Environments and Society, London School of Hygiene & Tropical Medicine, London, United Kingdom.

[2] Department of Infectious Disease Epidemiology, London School of Hygiene & Tropical Medicine, London, UK.

[3] AFRICSanté, Bobo-Dioulasso, Burkina Faso.

[4] Division of Child and Adolescent Health, Institute of Clinical Medicine, University of Oslo, Oslo, Norway

[5] Department of Pediatrics and Adolescent Health, University of Botswana, Gaborone, Botswana

[6] Department of Medicine, Clinical Epidemiology Division, Karolinska Institutet, Stockholm, Sweden

[7] Departement Biomédical et Santé Publique, Institut de Recherche en Sciences de la Santé, Ouagadougou, Burkina Faso.

[8] Wits Reproductive Health and HIV Institute, University of the Witwatersrand, Johannesburg, South Africa.

For peer review only

For peer review only - http://bmjopen.bmj.com/site/about/guidelines.xhtml

[9] Department of Population Health, Medical College, Aga Khan University, Nairobi, Kenya.

* Corresponding author email: cherie.part@lshtm.ac.uk. Address: Department of Public Health, Environments and Society, LSHTM, 15-17 Tavistock Place, London, WC1H 9SH.

For peer review only

For peer review only - http://bmjopen.bmj.com/site/about/guidelines.xhtml

ABSTRACT

Objective: To examine the effects of high ambient temperature on infant feeding practices and childcare.

Design: Secondary analysis of quantitative data from a prospective cohort study.

Setting: Community-based interviews in the commune of Bobo-Dioulasso, Burkina Faso. Exclusive breastfeeding is not widely practiced in Burkina Faso.

Participants: 866 women (1:1 urban:rural) were interviewed over 12 months. Participants were interviewed at three time points: cohort entry (when between 20 weeks gestation and 22 weeks postpartum), three and nine months thereafter. Retention at nine-month follow-up was 90%. Our secondary analysis focussed on postpartum women (n=857).

Exposure: Daily mean temperature (°C) measured at one weather station in Bobo-Dioulasso. Meteorological data were obtained from publicly available archives (TuTiempo.net).

Primary outcome measures: Self-reported time spent breastfeeding (minutes/day); exclusive breastfeeding of infants under six months (no fluids other than breast milk provided in past 24 hours); supplementary feeding of infants aged 6-12 months (any fluid other than breast milk provided in past 24 hours); time spent caring for children (minutes/day).

Results: The population experienced year-round high temperatures (daily mean temperature range=22.6–33.7°C). Breastfeeding decreased by 2.3 minutes/day (95% CI -4.6 to 0.04, p=0.05), and childcare increased by 0.6 minutes/day (0.06 to 1.2, p=0.03), per 1°C increase in same-day mean temperature. Temperature interacted with infant age to affect breastfeeding duration (p=0.02), with a stronger (negative) association between temperature and breastfeeding as infants aged (0–57 weeks). Odds of exclusive breastfeeding very young

For peer review only - http://bmjopen.bmj.com/site/about/guidelines.xhtml

infants (0-3 months) tended to decrease as temperature increased (OR 0.88, 0.75 to 1.02, p=0.09). There was no association between temperature and exclusive breastfeeding at 3-6 months or supplementary feeding (6-12 months).

Conclusions: Women spent considerably less time breastfeeding (~25 minutes/day) during the hottest, compared to coolest, times of the year. Climate change adaptation plans for health should include advice to breastfeeding mothers during periods of high temperature.

For peer review only

For peer review only - http://bmjopen.bmj.com/site/about/guidelines.xhtml

Strengths and limitations of this study

- This is the first study to quantify acute effects of ambient heat on breastfeeding behaviour.
- Multi-stage stratified sampling was used to select a population-representative cohort of pregnant and postpartum women in the commune of Bobo-Dioulasso, Burkina Faso.
- Detailed questionnaires enabled extensive confounder control.
- Outcome measures relied on self-reports, including time-use estimations; however questions were embedded within an extensive interview schedule, reducing the likelihood of response bias, and measures were used to assist participants with time estimations.
- The small sample size and short recruitment window may have limited our ability to detect statistically significant associations.

For peer review only

INTRODUCTION

Climate change is a growing threat to population health in Africa, [1, 2] with heatwaves increasing in severity and duration, especially in the Sahel. [3] Maternal and neonatal health will be affected through the adverse effects of heat on preterm birth, [4, 5] stillbirth, [4, 5] and maternal nutrition. [6] Child wasting and malnutrition are expected to increase. [2] High temperatures may also reduce cognitive function [7] and interfere with daily activities, leading to a decline in emotional health and wellbeing. [8] Mothers may find it difficult to breastfeed their infants under extreme heat, [9] and may also change their behaviour due to perceived risks to health. For example, there is still a common misconception among postpartum women in several African countries that breast milk is not sufficient to hydrate babies during hot weather; leading to supplementary feeding of infants, with sometimes not potable water, [10-13] and a reduction in exclusive breastfeeding. [10]

Breastfeeding and, in particular, exclusive breasfeeding has well-established benefits for child health and development. [14-16] Breastfeeding reduces the risk of diarrhoea and respiratory infections among infants, and is associated with a higher intelligence quotient and reduced obesity in later life. [16] There are also benefits for maternal health, with nursing mothers at lower risk of breast and, potentially, ovarian cancers. [16] The World Health Organization (WHO) recommends that infants are fed with breastmilk exclusively for the first six months and that no solids or other liquids are given during this period, including water. [17] However, the self-reported prevalence of exclusive breasfeeding is low in many African countries. [16, 18] In Burkina Faso, less than 25% of women reported exclusive breastfeeding of their young infants (less than six months old) in 2010-2015. [18]

It is not unusual for breastfeeding patterns to change in hot weather. Infants may refuse to feed during the hottest part of the day, or they may demand more frequent, but shorter, feeds

For peer review only - http://bmjopen.bmj.com/site/about/guidelines.xhtml

throughout the day. [19] In doing so, babies consume mostly low-fat milk (foremilk) and avoid breast milk with a high fat content (i.e. afternoon/evening milk and hindmilk). [19, 20] Mothers must change their breastfeeding patterns to accommodate their infants' needs, and may spend more time breastfeeding as temperatures rise. Conversely, women may spend less time breastfeeding during periods of high temperature due to increased discomfort for both mother and child, [9] increased provision of water (believed necessary to quench baby's thirst in some African settings), [21] and/or associated health effects, such as low energy [8] and heat exhaustion. [22]

Infants and young children are particularly vulnerable to heat injury and dehydration due to a greater surface area to body mass ratio. [23] Therefore, as temperatures rise in hot climates, mothers may spend more time watching over their children and other children in the household, keeping them hydrated, and tending to them when unwell. Such increased demands on time may cause difficulties for mothers in low-income countries such as Burkina Faso, where women work to supplement household income (particularly in agriculture, horticulture, and small trade) as well as undertaking important domestic responsibilities (including gathering food, water, fuel, and feeding livestock). [6] Most women work in the informal sector, [24] therefore paid maternity leave is uncommon and many women return to work early in the postpartum period.

Average monthly temperatures in Burkina Faso range between 25–33°C [25] and the impacts on infant care practices are largely unknown. Studies in South America, South Asia and Africa show seasonal differences in breastfeeding behaviour, with conflicting results. [26-29] For example, in Bihar, India, infants under six months were more likely to be exclusively breastfed in the cooler than warmer season. [29] Whereas, in rural Egypt, exclusive breastfeeding of infants aged 6–11 months was more prevalent in the hot than cool

For peer review only

For peer review only - http://bmjopen.bmj.com/site/about/guidelines.xhtml

season. [26] However, such studies are not sufficient to demonstrate an effect of ambient temperature as the competing time demands of women's domestic and agricultural workloads, [10, 30-33] as well as other potentially important drivers (e.g. household food security), also vary with season and weather in rural settings. [33, 34] With daily temperatures in West Africa expected to exceed 50°C in some regions, [35] further research is essential so that maternal and child health programmes can be updated.

This study aims to explore the effects of daily outdoor temperature on infant feeding practices and childcare in western Burkina Faso. We hypothesised that: (a) Time spent breastfeeding is associated with same-day temperature; (b) Women are less likely to breastfeed exclusively as temperatures rise; (c) Women are more likely to provide supplementary fluids as temperatures rise; (d) Time spent caring for children increases with same-day temperature.

METHODS

We undertook secondary analyses of quantitative data from an observational prospective cohort study of pregnant and postpartum women in Bobo-Dioulasso, Burkina Faso (the PopDev study), which aimed to assess the impacts of pregnancy on income- and non-income-generating activities among women in Burkina Faso, and to identify interventions to increase household income. [36]

Participants

Multi-stage stratified (urban vs. rural) sampling was used to select a population-representative cohort of pregnant and postpartum women in the commune of Bobo-Dioulasso. The 2006 census was used as the sampling frame to select locality clusters. It was estimated that each cluster must contain a minimum of 300-330 households to identify 30 eligible participants per cluster. Thirty-eight locality clusters were identified (14 urban, 24

For peer review only - http://bmjopen.bmj.com/site/about/guidelines.xhtml

rural), and participants were recruited within households at cluster-level. Households were visited per selected cluster using a modification of the World Health Organization's Expanded Program on Immunization sampling methodology.

Women were eligible to participate in the PopDev study if aged 15-45 years and between seven months gestation and three months postpartum at recruitment. [36] Sixty-two women did not meet the criteria for PopDev, but were retained in the dataset for secondary analyses. The full dataset comprised 866 women aged 14-47 years who were between 20 weeks gestation and 22 weeks postpartum at baseline. All women who completed at least one interview postpartum (*n*=857) were included in the secondary analysis (see figure 1).

Setting

The commune of Bobo-Dioulasso is predominantly urban, and includes the second largest city in Burkina Faso (Bobo-Dioulasso) with approximately 900,000 inhabitants. [37] Small settlements and villages, with a mainly agricultural focus, are located in rural areas surrounding the large urban centre. All rural participants in this study resided within 40-50 km of the city.

The commune has a tropical savannah climate, [38] with two distinct seasons; dry (November–May) and rainy (June–October). During the dry season, average temperatures are highest in March–May and lower in November–February.

Data collection

Participants were interviewed in their homes at three time points: cohort entry, and three and nine months thereafter. Retention at the nine-month visit was 90%. Several attempts were made to interview each woman at each interview round. The reason for non-interview was recorded, when possible. Eight hundred and 39 participants were recruited immediately

For peer review only - http://bmjopen.bmj.com/site/about/guidelines.xhtml

before/during the first round of interviews (29 November 2013 – 23 March 2014). Twenty-seven women were recruited during the second or third round of interviews (04 March 2014 – 09 September 2014; 02 September 2014 – 12 November 2014, respectively). All interviews occurred between 29 November 2013 and 12 November 2014, using structured questionnaires to ensure the same wording of questions for all participants. Interviews were conducted in local languages (predominantly Dioula) and homogeneity in translations was verified during interviewer training. All questions were designed to be non-leading.

During each interview, participants were asked to recall their activities on the previous day (or two days previous when the day before interview was atypical) and how many minutes they had spent on each activity. The recall period was defined as, "between waking up yesterday morning and waking up this morning". Participants described their activities and the interviewer categorised them using a pre-defined list, which was added to when needed. Breastfeeding, caring for children (bathing/dressing, feeding, playing/watching, tending to when unwell), income-generating work (e.g. agro-processing for trade, sale of products at market, small business, office activities), attending classes (e.g. literacy courses), and household chores (e.g. preparing meals, cleaning clothes, washing dishes, fetching water, fetching fuel) were included in this list.

To assist participants in time-use recall, women were initially asked to list all activities that they had engaged in during a specific time of day (e.g. between waking up and midday). Participants were then asked probing questions to assist them in estimating the duration of each activity. For example, some women said that they woke with the call of the muezzin, which enabled the interviewer to determine time of wake. Participants would then be asked if they began their first listed activity immediately, or if they did something else first. The interviewer asked when their first activity ended to which women responded, "to the first rays

For peer review only

For peer review only - http://bmjopen.bmj.com/site/about/guidelines.xhtml

181 of sunshine", for example. Thus, the interviewer had adequate information to estimate time
182 duration of the first activity. Once participants had finalised their time-use estimates for the
183 specified period, the process was repeated for the next time of day, until the full 24-hour
184 recall period was complete. Participants used a notebook to draft and revise time-use
185 estimates before final responses were recorded in the questionnaire. Indications of time
186 (particularly the path of the sun), combined with current events of life, served as benchmarks
187 for time estimations.

188 At first interview following childbirth (interview round one or two), participants were asked
189 how many children were born and the date of delivery. At interview rounds two and three,
190 women were asked if they were still breastfeeding their baby; if their baby had anything else
191 to drink in the past 24 hours; and which (if any) fluids had their baby been given to drink in
192 the past 24 hours. These questions were used to construct binary study outcomes of exclusive
193 breastfeeding (still breastfeeding and no fluids other than breast milk provided in past 24
194 hours) and supplementary feeding (any fluids other than breast milk provided in past 24
195 hours).

196 Interviews included additional questions to those described above in order to fulfil the aims
197 of the PopDev study. Each interview lasted approximately 45-60 minutes. Questionnaires are
198 available at: https ://datacompass.lshtm.ac.uk/id/eprint/64/.

199 **Meteorological data**

200 Daily meteorological data (mean, minimum and maximum temperature [°C], relative
201 humidity [%], and windspeed [km/h]) were obtained from TuTiempo.net [39] for a single
202 weather station in Bobo-Dioulasso, located in the industrial district (Zone Industrielle)
203 (11°09'36.0"N 4°18'36.0"W). Eleven days of temperature data were missing over the study
204 period and were excluded from the analysis.

For peer review only - http://bmjopen.bmj.com/site/about/guidelines.xhtml

Outcomes

Four outcomes were assessed: (1) Breastfeeding duration: self-reported time spent breastfeeding on the day/night before interview (total minutes in 24 hours); (2) Exclusive breastfeeding: no liquids other than breast milk given in past 24 hours; (3) Supplementary feeding: any liquid other than breast milk given in past 24 hours; and (4) Childcare duration: self-reported time spent exclusively on childcare (including bathing/dressing, feeding, playing/watching, tending to when unwell) on the day/night before interview (total minutes in 24 hours). Breastfeeding duration, exclusive breastfeeding, and supplementary feeding outcomes referred specifically to the target (newborn) infant. Childcare duration did not refer specifically to the newborn infant.

Exposure

The primary exposure was daily mean temperature (°C). Daily mean temperature correlated strongly with daily minimum (r=0.8, p<0.001) and maximum temperatures (r=0.89, p<0.001) and was considered the best approximation of overall exposure during the recall period. Apparent daily mean temperature (°C) was calculated from daily mean temperature, relative humidity and windspeed, using the R HeatStress package, [40] to test the robustness of our findings.

Data analyses

Daily exposures were linked with outcomes at individual level, by date of interview minus one-day (t-1) to reflect same-day temperature when activities were undertaken. Categorical outcomes, potential confounding variables and covariates (see table S1) were summarised as proportions (expressed as percentages). Continuous variables were summarised as mean ±

For peer review only - http://bmjopen.bmj.com/site/about/guidelines.xhtml

standard deviation (SD) if normally distributed, or as median and interquartile range if not normally distributed. Summary statistics were stratified by interview round where applicable.

The functional form of temperature-time use (breastfeeding, childcare) associations were determined by aggregating outcome data to daily level, fitting natural cubic splines of time (to adjust for seasonal patterns and trends unrelated to temperature), and examining locally-weighted smoothing of Pearson's standardised residuals from the fitted splines, plotted against daily mean temperature.

Multilevel linear regression was then used to estimate the effects of daily mean temperature on time spent (i) breastfeeding, and (ii) caring for children. This approach made use of all available time-use data on breastfeeding and childcare, while accounting for the longitudinal and nested structure of these data. Interview contacts (level one) were nested within individual participants (level two), nested within the locality clusters from which the population was sampled (level three). Each level was defined as a random coefficient with random intercept to allow for correlation within-individuals and clusters. A first-order autoregressive correlation structure allowed for unequal spacing of interviews.

Separate models were developed for each outcome, and adjusted for interview round. Indicator terms were included for calendar month of interview to adjust for season and long-term trends. Adjusting for month (rather than season) of interview provided tighter control of possible confounders, such as household food security and fasting during Ramadan. Other covariates (number born [singleton; multiple birth], infant age [weeks], maternal age [≤ 19; 20–34; ≥ 35 years], gravidity [1; 2–5; ≥ 6 pregnancies], residential area [urban; rural], living arrangements [with partner full-time; with partner periodically; not with partner; not in a relationship], paid work or education [minutes/day], domestic work [minutes/day], and roofing materials [natural; rudimentary; contemporary]; see table S1) were added to the

For peer review only - http://bmjopen.bmj.com/site/about/guidelines.xhtml

models one-by-one, following a forward stepwise process, and were retained in the model if they were significantly associated with the outcome ($p<0.05$), improved model fit (reduced the Akaike Information Criterion by ≥2%), and/or changed the temperature effect by ≥10%. Cases with missing data were excluded from the analysis. Participants lost to follow-up were included in the analysis.

We restricted our analyses of exclusive breastfeeding and supplementary feeding to infants aged less than six months and 6-12 months, respectively. This follows from WHO's recommendations that infants are breastfed exclusively for the first six months of life, and that supplementary foods are only introduced thereafter. [17] Data were available for two time points (interview rounds two and three). To reduce model complexity, these outcomes were analysed cross-sectionally, at single time points: Exclusive breastfeeding at interview round two, and supplementary feeding at interview round three, based on the age range of infants at each round.

Logistic regression was used to test for associations between mean temperature and the odds of (i) exclusive breastfeeding and (ii) supplementary feeding, adjusting for month of interview, and other important confounders and covariates following the same process described above. As the effects of temperature on exclusive breastfeeding may change as infants age, [29] our exclusive breastfeeding analysis was age-stratified (< 3 months; 3 to < 6 months). Interactions between mean temperature and: (i) infant age; (ii) urban/rural residence; and (iii) roofing materials of the home were tested in all models (both multilevel and logistic).

Sensitivity analyses involved re-specifying models with: (i) alternative levels of seasonal control (indicator variables for 'season' rather than 'month'; natural cubic splines of calendar time with three knots); and (ii) apparent, rather than observed, daily mean temperature.

For peer review only

For peer review only - http://bmjopen.bmj.com/site/about/guidelines.xhtml

Analyses were done in R 4.0.4, [41] using RStudio and the following R packages: lme4; [42] nlme; [43] stats; [41] splines; [41] effects; [44] ggplot2; [45] HeatStress; [40] Hmisc. [46]

Patient and public involvement

Stakeholders were involved during development of the original proposal in June 2012. The objectives and plans for the primary (PopDev) study were discussed with representatives from the community and reproductive health NGOs, as well as health professionals and policy makers at local and national levels, by means of a workshop, email, and telephone.

Stakeholders from the policy or associative arena presented their policies at the workshop to inform a group discussion on how best the study objectives could respond to their information needs. In another exercise, stakeholders were asked to identify one positive, one negative, and one surprising thing about the proposed study, which yielded particularly useful information when developing the proposal. We received feedback on substantive and methodological aspects of the project, and on communication issues, which was used to shape the study objectives and methodologies.

The interview schedule was piloted with members of the community. Feedback on interview duration, meaningfulness and clarity of questions, and perceived gaps, was used to refine the wording of questions and to add/remove items. At the end of the primary (PopDev) study, a stakeholder consultation workshop took place to discuss the findings and their implications for cross-sectoral interventions, involving policy makers from different ministries and NGO staff.

Pregnant and postpartum women, as well as community members, in the Kaya and Bogodogo health districts of Burkina Faso were involved before the secondary study began. In-depth interviews with pregnant and postpartum women (n=40), and focus group discussions with

For peer review only - http://bmjopen.bmj.com/site/about/guidelines.xhtml

community members, were undertaken in October–November 2020. [9] The objectives for the secondary analysis were developed and informed by the lived experiences of postpartum women reported during this qualitative work. Specifically, women described how hot weather impedes breastfeeding due to excessive sweating and the discomfort of both mothers and their babies. [9]

Qualitative findings were discussed with stakeholders in maternal and neonatal health, climate change adaptation, as well as pregnant and postpartum women and community members, during a co-design workshop in Ouagadougou, Burkina Faso. Here, breastfeeding messaging was highlighted as an important area of focus for future research and interventions aimed at reducing the impact of high temperatures on childbearing women and their newborn infants. We will continue our engagement with community members in the Kaya and Bogodogo health districts and will disseminate our findings through meetings, written summaries, and audio-visual materials. We will also engage with health decision-makers and provide summaries of the evidence and targeted policy briefs to support decision-makers in actions to reduce the impact of extreme high temperatures on maternal and neonatal health.

RESULTS

The population experienced year-round high temperatures, with an intra-annual range in daily mean temperature of 22.6–33.7°C. Figure S1 (supplementary material) shows daily minimum and maximum temperatures throughout the study period.

Eight-hundred and fifty-seven participants birthed 881 children (833 singleton births; 24 twin births). Six stillbirths, eight deaths in live born children, and 18 deaths in infants of unknown status at birth were reported. The mean age of women at recruitment was 26.9 years (SD=6.2 years), with a median gravidity of 3 pregnancies (IQR=2–5 pregnancies). Only 33 women

For peer review only - http://bmjopen.bmj.com/site/about/guidelines.xhtml

Table 1. Cohort characteristics, activities, and average daily temperature at each interview round.

	Interview round		
	1	2	3
Total interviewed (N)	839	810	792
Cohort characteristics			
*% urban [N] (NA)**	49.9 [419] (0)	49.3 [390] (19)	49.7 [389] (10)
% postpartum [N]	49.5 [415]	100 [810]	100 [792]
*% working in informal sector [N] (NA)**	37.6 [315] (1)	42.5 [343] (3)	48.9 [386] (2)
Living arrangements			
% with partner full-time [N]	80.9 [679]	80.1 [649]	80.1 [634]
% with partner periodically [N]	6.9 [58]	6.4 [52]	5.3 [42]
% not living with partner [N]	2.5 [21]	4.3 [35]	3.2 [25]
% not in a relationship [N]	0 [0]	8.8 [71]	7.7 [61]
% unknown [N]	9.7 [81]	0.4 [3]	3.8 [30]
Postpartum women only			
*% breastfeeding [N] (NA)**	100 [408] (7)	99.7 [782] (26)	99.7 [765] (25)
*% supplementary feeding [N] (NA)**		80.2 [628] (27)	98.0 [752] (25)
Time use (self-reported minutes/day)			
*Breastfeeding (median [IQR]) (NA)**	120 [80–180] (11)	180 [120–180] (23)	240 [121–240] (26)
*Childcare (median [IQR]) (NA)**	30 [15–40] (5)	30 [20–40] (11)	20 [15–30] (21)
Paid work/education (median [IQR])	0 [0–0]	0 [0–92]	300 [0–420]
Domestic work (median [IQR])	180 [110–240]	215 [145–300]	180 [130–235]
Infant age (weeks) (median [IQR]) *(NA)**	5.7 [2.6–9.9] (17)	12.6 [6.4–19.0] (25)	33.9 [27.1–41.0] (31)
Daily mean temperature (°C) (median [range]) *(NA)**	27.9 [22.7–32.8] (0)	27.0 [22.9–33.7] (11)	27.2 [23.3–30.3] (0)

Number (N) of women and % of total interviewed at each survey round, or summary statistics specified.

* Missing values were excluded from calculations. NA = N missing. Where NA is not provided, N=0.

(3.9%) were formally employed at baseline, of which 21 women were eligible for (or benefiting from) maternity leave. Informal paid work was more common (see table 1). Most

For peer review only - http://bmjopen.bmj.com/site/about/guidelines.xhtml

women (808 of 839 interviewed at baseline) lived in houses with contemporary roof materials; primarily sheet metal (731 women) and timber (81 women).

Median time between first and second interviews was 92 days (IQR 80–108 days), and 149 days (IQR 141–155 days) between the second and third interviews. Total median follow-up time was 236 days (IQR 227–257 days). One woman refused to participate during the second round of interviews and one woman was travelling during the third round. The reasons why other women were lost to follow-up (n=74) are unknown.

The vast majority of postpartum women reported breastfeeding their infants at each interview round (table 1). However, the incidence of exclusive breastfeeding was low. Only 148 of 710 infants (20.8%) aged less than six months were exclusively breastfed on the day/night before interview two, and only 11 of 157 (7%) were exclusively breastfed before interview three.

On average, daily breastfeeding duration increased over time (figure 2a). After adjusting for long-term trends, a slight decrease in breastfeeding duration was observed as temperatures increased (figure 3a). Before adjusting for potential confounders (accounting only for the longitudinal and nested structure of the data), breastfeeding was estimated to decrease by 5.6 minutes/day (95% CI -7.0 to -4.1, p<0.001, n=783 women) per 1°C increase in same-day mean temperature. After controlling for important confounders (interview round, month of interview, singleton/multiple birth, residential area, and time spent on paid work or education [minutes/day]), breastfeeding was estimated to decrease by 2.3 minutes/day (95% CI -4.6 to 0.04, p=0.05, n=783 women) per 1°C increase in same-day mean temperature (table S2). This estimate was for infants aged 0.6–57 weeks (median=18.6 weeks). However, temperature interacted with infant age to affect breastfeeding duration (p=0.02). Time spent breastfeeding very young infants (four weeks) did not change with temperature. As infants aged, women were predicted to spend increasingly less time breastfeeding at high temperatures (figure 4).

For peer review only - http://bmjopen.bmj.com/site/about/guidelines.xhtml

On average, women spent less time on exclusive childcare at interview three (table 1), which coincided with the rainy and early dry/cooler seasons (figure 2b). After seasonal control, a slight increase in childcare time was observed as temperatures increased (figure 3b). Before adjustment, we estimated a 0.4-minute increase (0.1 to 0.8, p=0.02, n=814 women) in daily childcare per 1°C increase in mean temperature. We estimated a 0.6-minute increase (0.06 to 1.2, p=0.03, n=787 women) in childcare per 1°C increase in temperature after adjusting for interview round, calendar month, singleton/multiple birth, infant age, maternal age, women's living arrangements, and time spent on paid work or education (minutes/day) (table S3).

There was suggestive evidence that very young infants (< 3 months) were less likely to be exclusively breastfed as temperatures increased (unadjusted OR=0.88, 0.76 to 1.02, p=0.08, n=338; adjusted OR=0.88, 0.75 to 1.02, p=0.09, n=331). Whereas, there was no evidence that daily mean temperature affected the odds of exclusive breastfeeding at 3-6 months, either before (OR=0.98, 0.80 to 1.20, p=0.8, n=237) or after adjustment (OR=1.13, 0.86 to 1.52, p=0.4, n=235). Variability in daily mean temperature was similar for both groups (< 3 months = 24.1–33.7°C; 3-6 months = 25.3–33.7°C), however the rate of exclusive breastfeeding was higher among women with younger (< 3 months = 30% [120 of 396 women]) than older infants (3-6 months = 9% [28 of 312 women]).

A large proportion of women provided supplementary fluids to their infant (table 1); primarily water, herbal tea and, in the rainy season, boiled water. Milk other than breastmilk was rarely given. By 6-12 months, the provision of supplementary fluids was almost universal (99.2% [605 of 610] infants). Therefore, analysis of association with daily mean temperature was not feasible.

There was no evidence of an interaction between temperature and residential area (urban/rural) or type of roofing materials on any outcome measured (p>0.05).

For peer review only - http://bmjopen.bmj.com/site/about/guidelines.xhtml

Estimated temperature effects were generally robust to sensitivity analyses. The main effect on breastfeeding duration was very robust and increased in statistical significance with alternative methods of seasonal control. The interaction effect between temperature and infant age on breastfeeding duration, and the main effect of temperature on daily childcare duration, were also fairly robust but with reduced significance. Redefining the exposure as apparent (“feels-like”) daily mean temperature did not change the estimated effect on exclusive breastfeeding of very young infants (< 3 months), but increased the statistical significance of this finding.

DISCUSSION

This study explored the impacts of high ambient temperature on infant feeding practices among postpartum women in a low-income setting. We found a decrease in breastfeeding duration as temperatures increased; approximating to a 25-minute reduction in breastfeeding on the hottest, compared to coolest, days of the year. The extent of this impact largely depended on the age of the infant. From approximately four months onwards, we predicted an increasingly negative impact of high temperature on breastfeeding duration. For younger infants, temperature had a lower impact. However, there was suggestive evidence that very young infants (under three months) were less likely to be breastfed exclusively as temperature increased.

There may be several explanations for the reduction in breastfeeding duration as temperatures rise in hot climates. Infants may demand less milk in order to limit heat-generation, or they may become too uncomfortable to feed. [9] On the other hand, mothers may offer less breast milk due to their own discomfort under very hot conditions [9, 47] and/or due to a misperception that babies require supplementary water, especially on hot days. [10, 12, 13]

For peer review only - http://bmjopen.bmj.com/site/about/guidelines.xhtml

Despite efforts to improve breastfeeding practices in sub-Saharan Africa, [48] the belief that breast milk is insufficient to hydrate babies and that water is needed to quench their thirst still prevails in several societies. [12, 49-51] In the commune of Bobo-Dioulasso, provision of supplementary fluids (particularly water) was widespread. More than 95% of infants aged three months and older were given non-milk fluids in the 24 hours before interview. It is recommended that infants under six months are breastfed more often in hot weather [52] and, ideally, the total intake of breast milk over a 24-hour period would increase to avoid infant dehydration. However, our findings tentatively suggest the contrary. Rather than increase breastfeeding to prevent infant dehydration, the women in our study may have provided a greater volume of supplementary fluids when temperatures increased.

The hot climate has been identified as a barrier to exclusive breastfeeding in the Democratic Republic of the Congo, [10] southern Zimbabwe, [13] Ghana, [12], and Ethopia [49], and might (at least, partially) explain the very low rate of exclusive breastfeeding found herein. In the cooler sub-tropical climate of Bihar, India, odds of exclusive breastfeeding was significantly lower in summer than in winter or transitional seasons. Perceived thirst was proposed as an underlying cause for the higher rates of supplementary feeding in warmer months. [29] However, in contrast to our findings, the impact of season was greater for infants aged 3-6 months than for infants under 3 months. [29] The warmer climate of Bobo-Dioulasso, cultural values and beliefs around breastfeeding, [53] and the comparatively low rate of exclusive breastfeeding in our study (8% vs. 70% of infants aged 3-6 months) likely explain this discrepancy in findings.

Many women across the world report perceived inadequacy of milk supply as their main reason for early weaning. [54] It is not clear if breast milk production is impacted by heat exposure, either directly or indirectly. High temperatures may exacerbate water stress,

For peer review only - http://bmjopen.bmj.com/site/about/guidelines.xhtml

increasing the risk of dehydration among mothers in water-poor regions. However, the effects of maternal dehydration on breast milk production in hot climates are largely unknown. [55, 56] Several studies have shown that exclusively breastfed infants maintain normal hydration under hot conditions, [57-59] indicating that the quantity of breast milk is not affected. However, field and experimental animal studies have shown that both the yield and nutritional composition of ruminant milk decline under hot conditions. [60] Further, the milk production capacity of animal mammary epithelial cells was found to decline following in vitro exposure to high temperatures (41°C). [61] To our knowledge, no studies have examined breast milk production in relation to temperature, but there is evidence that maternal stress affects breast milk composition [62] and delays secretory activation. [63] Even if milk supply is not adversely affected, high temperatures may contribute to women's perception of inadequate milk supply.

The marginal increase in exclusive childcare time with temperature is not easily explainable given the range of activities included in this outcome (e.g. bathing/dressing, playing/watching, tending to when unwell). Tasks, such as dressing children, may take longer under hot conditions due to excessive sweating and/or low energy levels. Increased effort may be required to bathe or soothe children when temperatures rise, or women might spend more time monitoring other children in the household.

The main strengths of this study are the longitudinal dataset, population-based sampling, detailed questionnaire on activities, small loss to follow-up, and extensive confounder control. The main limitations are the small sample size, which may have reduced our ability to detect statistically significant associations, and the short recruitment window (ideally, the study would have been conducted over several years). However, 2014 was a climatically typical year in Burkina Faso during 2010s. [25] We used meteorological data recorded at one

For peer review only - http://bmjopen.bmj.com/site/about/guidelines.xhtml

weather station in Bobo-Dioulasso, located in the urban centre. We could not assign exposures to women's residential addresses, but daily variability in temperature exposures were likely to be consistent over the study area, even if absolute temperatures varied slightly.

Measurements of breastfeeding and childcare duration relied on self-reported time-use estimations. We put several measures in place to assist participants, including the recent and short (24-hour) recall period with questions aimed at establishing a 24-hour timeline. However, it is possible that temperature affected participants' ability to estimate time, despite the use of benchmarks (e.g. path of the sun). Time-use diaries and direct observation offer arguably more robust methods for future research, although each has limitations. Our measurement of exclusive breastfeeding was not optimal, but women were not asked directly if they breastfed their infant exclusively. Instead, this outcome was constructed from women's recall of all fluids given to their child in the past 24 hours. Questions on infant feeding practices and childcare were embedded within an extensive interview schedule, further reducing the possibility of response bias. Finally, the outcome of childcare is complex and refers to time spent with children of all ages as this question was not specifically phrased to indicate the target (newborn) child.

Larger studies are needed to further examine the impacts of heat on infant feeding practices in hot climates. Future research should consider temperature in relation to the number and duration of individual breastfeeds, and to the volume of breast milk and supplementary fluids consumed by infants, over a 24-hour period. Research should also seek to determine if high temperatures impact on breast milk production. Actions should be taken to ensure that hot weather does not negatively impact on breastfeeding behaviour. Effective interventions are likely to require a multidimensional approach. [64] It is important that health workers and

For peer review only

For peer review only - http://bmjopen.bmj.com/site/about/guidelines.xhtml

mothers are informed about normal heat-induced changes in infant breastfeeding patterns so that such changes are not misinterpreted as a need for supplementation.

CONCLUSIONS

Exclusive breastfeeding is an essential cornerstone for the wellbeing and survival of infants. Our findings suggest a substantial decrease in breastfeeding duration, and potentially lower odds of exclusive breastfeeding very young infants, during hot weather. These findings are important as infants require increased hydration to cope physiologically with increased heat, and the safest form of hydration for young infants is breast milk.

Larger studies are needed in Burkina Faso and beyond as climate change in Africa is accelerating. [65] Without effective interventions, mothers may find it increasingly difficult to breastfeed their infants as temperatures rise. Maternal and child health programmes in hot climates should be updated to improve messaging and breastfeeding practices during extreme hot weather.

For peer review only

For peer review only - http://bmjopen.bmj.com/site/about/guidelines.xhtml

Acknowledgements

We thank Maurice Yaogo, Patrick Ilbouldo, André Soubeiga, Katerini Storeng, Denis Ouedraogo, Seydou Drabo, and Tim Powell-Jackson for their valuable contribution to the primary (PopDev) study. We also gratefully acknowledge all women who participated in the PopDev study.

Contributors

VF and SKovats conceived the study (secondary analysis). JAC and RG collected data. CP conducted the statistical analysis under the supervision of SH. All authors (CP, VF, JAC, RG, SH, BN, NR, KK, MC, AL, SK and SKovats) interpreted the results. CP drafted the paper. All authors critically revised the paper for important intellectual content and approved the final version for publication. All authors agreed to be accountable for all aspects of the work.

Funding

The PopDev study was supported by the Economic and Social Research Council (ESRC) in response to the Joint ESRC-WOTRO-RCN-PRB-Hewlett call on Population & Development [grant number ES/K011049/1]. The secondary analysis was supported by the Natural Environment Research Council (NERC) [grant numbers NE/T013613/1, NE/T01363X/1]; Research Council of Norway (RCN) [grant number 312601]; and The Swedish Research Council for Health, Working Life and Welfare in collaboration with the Swedish Research Council (Forte) [grant number 2019-01570]; coordinated through a Belmont Forum partnership.

Competing interests

None declared.

For peer review only

504 **Ethics approval**

505 The study was approved by the Research Ethics Committees of Centre Muraz, Burkina Faso
506 (reference: A16-2013/CE-CM) and the London School of Hygiene & Tropical Medicine,
507 United Kingdom (reference: 6401). The free and informed consent of each participant was
508 obtained before each interview.

509 **Data sharing statement**

510 Individual-level deidentified participant data from the primary (PopDev) study are available
511 to researchers who have a valid research question, which is not being investigated by the
512 primary research team. A data sharing agreement will be required. All data requests should be
513 made via https://datacompass.lshtm.ac.uk/id/eprint/64/. The questionnaires, consent form,
514 data dictionary (codebook), and user guide are publicly available from
515 https://datacompass.lshtm.ac.uk/id/eprint/64/. Data have been available from 2018 with no
516 end date. Meteorological data are publicly available from TuTiempo
517 (https://www.tutiempo.net/). Statistical code is available from the corresponding author.

For peer review only

For peer review only - http://bmjopen.bmj.com/site/about/guidelines.xhtml

REFERENCES

1 Opoku SK, Filho WL, Hubert F, et al. Climate change and health preparedness in Africa: Analysing trends in six African countries. *Int J Environ Res Public Health* 2021;18:4672 doi: 10.3390/ijerph18094672.
2 Baker RE, Anttila-Hughes J. Characterizing the contribution of high temperatures to child undernourishment in Sub-Saharan Africa. *Sci Rep* 2020;10:18796–96 doi: 10.1038/s41598-020-74942-9.
3 Sambou M-JG, Pohl B, Janicot S, et al. Heat waves in spring from Senegal to Sahel: Evolution under climate change. *Int J Climatol* 2021;41:6238–53 doi: 10.1002/joc.7176.
4 Chersich MF, Pham MD, Areal A, et al. Associations between high temperatures in pregnancy and risk of preterm birth, low birth weight, and stillbirths: systematic review and meta-analysis. *BMJ* 2020;371:m3811 doi: https://doi.org/10.1136/bmj.m3811.
5 McElroy S, Ilango S, Dimitrova A, et al. Extreme heat, preterm birth, and stillbirth: A global analysis across 14 lower-middle income countries. *Environ Int* 2022;158:106902 doi: 10.1016/j.envint.2021.106902 [published Online rst: 20211006].
6 Romero González AM, Belemvire A, Saulière S. Climate Change and Women Farmers in Burkina Faso: Impact and adaptation policies and practices. Oxfam Research ReportOxford, UK: Oxfam; 2011.
7 Saini R, Srivastava K, Agrawal S, et al. Cognitive deficits due to thermal stress: An exploratory study on soldiers in deserts. *Med J Armed Forces India* 2017;73:370-74 doi: 10.1016/j.mjafi.2017.07.011 [published Online rst: 20170906].
8 Tawatsupa B, Yiengprugsawan V, Kjellstrom T, et al. Heat stress, health and well-being: findings from a large national cohort of Thai adults. *BMJ Open* 2012;2:e001396 doi: 10.1136/bmjopen-2012-001396.
9 Kadio K, Mariam C, Scorgie F, et al. The experience of heat stress among pregnant and postpartum women and their neonates in Burkina Faso: a qualitative analysis. *Trop Med Int Health* 2021;26:253–54. [Special Issue: Abstracts of the 12th European Congress on Tropical Medicine and International Health, 28 September - 1 October 2021, Bergen, Norway] doi: 10.1111/tmi.13633.
10 Burns J, Emerson JA, Amundson K, et al. A qualitative analysis of barriers and facilitators to optimal breastfeeding and complementary feeding practices in South Kivu, Democratic Republic of Congo. *Food Nutr Bull* 2016;37:119–31 doi: 10.1177/0379572116637947.
11 Cresswell JA, Ganaba R, Sarrassat S, et al. Predictors of exclusive breastfeeding and consumption of soft, semi-solid or solid food among infants in Boucle du Mouhoun, Burkina Faso: A cross-sectional survey. *PLOS ONE* 2017;12:e0179593 doi: 10.1371/journal.pone.0179593.
12 Nsiah-Asamoah C, Doku DT, Agblorti S. Mothers' and Grandmothers' misconceptions and socio-cultural factors as barriers to exclusive breastfeeding: A qualitative study involving Health Workers in two rural districts of Ghana. *PLOS ONE* 2020;15:e0239278 doi: 10.1371/journal.pone.0239278.
13 Mundagowa PT, Chadambuka EM, Chimberengwa PT, et al. Barriers and facilitators to exclusive breastfeeding practices in a disadvantaged community in southern Zimbabwe: A maternal perspective. *World Nutrition* 2021;12:73–91 doi: 10.26596/wn.202112173-91.
14 Walters DD, Phan LTH, Mathisen R. The cost of not breastfeeding: global results from a new tool. *Health Policy Plan* 2019;34:407–17 doi: 10.1093/heapol/czz050.
15 Wells JC, Sawaya AL, Wibaek R, et al. The double burden of malnutrition: aetiological pathways and consequences for health. *Lancet* 2020;395:75–88 doi: 10.1016/S0140-6736(19)32472-9.
16 Victora CG, Bahl R, Barros AJD, et al. Breastfeeding in the 21st century: epidemiology, mechanisms, and lifelong effect. *Lancet* 2016;387:475–90 doi: 10.1016/S0140-6736(15)01024-7.
17 World Health Organization. Infant and Young Child Feeding. 2021. https://www.who.int/en/news-room/fact-sheets/detail/infant-and-young-child-feeding (accessed 10 August 2021).
18 Issaka AI, Agho KE, Renzaho AM. Prevalence of key breastfeeding indicators in 29 sub-Saharan African countries: a meta-analysis of demographic and health surveys (2010–2015). *BMJ Open* 2017;7:e014145 doi: 10.1136/bmjopen-2016-014145.

For peer review only

For peer review only - http://bmjopen.bmj.com/site/about/guidelines.xhtml

19 Australian Breastfeeding Association. Breastfeeding through warmer weather. 2017. https://www.breastfeeding.asn.au/bf-info/you-and-your-breastfed-baby/cool (accessed 08 Nov 2021).
20 Ballard O, Morrow AL. Human milk composition: nutrients and bioactive factors. *Pediatr Clin North Am* 2013;60:49–74 doi: 10.1016/j.pcl.2012.10.002.
21 Bhattacharjee NV, Schaeffer LE, Marczak LB, et al. Mapping exclusive breastfeeding in Africa between 2000 and 2017. *Nature Medicine* 2019;25:1205-12 doi: 10.1038/s41591-019-0525-0.
22 Adegebo BO. Urban thermal perception and self-reported health effects in Ibadan, south west Nigeria. *Int J Biometeorol* 2022;66:331-43 doi: 10.1007/s00484-021-02168-z.
23 Stanberry LR, Thomson MC, James W. Prioritizing the needs of children in a changing climate. *PLoS Med* 2018;15:e1002627 doi: 10.1371/journal.pmed.1002627 [published Online rst: 20180731].
24 Weber M. Burkina Faso Jobs Diagnostic: Overview and Suggestions for a Strategic Framework for Jobs. Jobs Series;15. Washington DC, USA: World Bank Group; 2018. https://openknowledge.worldbank.org/handle/10986/31033 (accessed 15 December 2021).
25 World Bank Group. Climate Change Knowledge Portal. Burkina Faso. Current Climate. 2021. https://climateknowledgeportal.worldbank.org/country/burkina-faso/climate-data-historical (accessed 20 Oct 2021).
26 Serdula MK, Seward J, Marks JS, et al. Seasonal differences in breast-feeding in rural Egypt. *Am J Clin Nutr* 1986;44:405–09 doi: 10.1093/ajcn/44.3.405.
27 Hossain MM, Radwan MM, Arafa SA, et al. Prelacteal Infant Feeding Practices in Rural Egypt. *J Trop Pediatr* 1992;38:317–22 doi: 10.1093/tropej/38.6.317.
28 González-Chica DA, Gonçalves H, Nazmi A, et al. Seasonality of infant feeding practices in three Brazilian birth cohorts. *Int J Epidemiol* 2012;41:743–52 doi: 10.1093/ije/dys002.
29 Das A, Chatterjee R, Karthick M, et al. The influence of seasonality and community-based health worker provided counselling on exclusive breastfeeding - findings from a cross-sectional survey in India. *PLOS ONE* 2016;11:e0161186 doi: 10.1371/journal.pone.0161186.
30 Nankumbi J, Muliira JK. Barriers to infant and child-feeding practices: a qualitative study of primary caregivers in Rural Uganda. *J Health Popul Nutr* 2015;33:106–16.
31 Matare C, Craig H, Martin S, et al. Barriers and opportunities for improved exclusive breast-feeding practices in Tanzania: Household trials with mothers and fathers. *Food Nutr Bull* 2019;40:308-25 doi: 10.1177/0379572119841961.
32 Moyo G, Magaisa T, Pagiwa A, et al. Barriers and facilitators of exclusive breastfeeding: Findings from a barrier analysis conducted in Mwenezi and Chiredzi Districts, Zimbabwe. *World Nutrition* 2020;11:12–21 doi: 10.26596/wn.202011312-21.
33 Randell H, Grace K, Bakhtsiyarava M. Climatic conditions and infant care: implications for child nutrition in rural Ethiopia. *Popul Environ* 2021;42:524–52 doi: 10.1007/s11111-020-00373-3.
34 Sorgho R, Mank I, Kagoné M, et al. “We will always ask ourselves the question of how to feed the family”: Subsistence farmers’ perceptions on adaptation to climate change in Burkina Faso. *IJERPH* 2020;17:7200.
35 Fitzpatrick RGJ, Parker DJ, Marsham JH, et al. How a typical West African day in the future-climate compares with current-climate conditions in a convection-permitting and parameterised convection climate model. *Clim Change* 2020;163:267–96 doi: 10.1007/s10584-020-02881-5.
36 Cresswell J, Drabo S, Filippi V, et al. Productivity, family planning and reproductive health in Burkina Faso: the PopDev study. London, UK: London School of Hygiene & Tropical Medicine, 2015.
37 Institut National de la Statistique et de la Démographie (INSD). Cinquième Recensement Général de la Population et de l’Habitation du Burkina Faso: Résultats Préliminaires. 2020. https://www.insd.bf/contenu/documents_rgph5/RAPPORT_PRELIMINAIRE_RGPH_2019.pdf (accessed 15 August 2021).
38 Beck HE, Zimmermann NE, McVicar TR, et al. Present and future Köppen-Geiger climate classification maps at 1-km resolution. *Sci Data* 2018;5:180214 doi: 10.1038/sdata.2018.214.
39 TuTiempo. Clima Bobo-Dioulasso. Datos climáticos: 1973 - 2021. https://www.tutiempo.net/clima/ws-655100.html, 2021.
40 Ana, MeteoSwiss. anacv/HeatStress: zenodo (v1.0.7_zenodo) [program]. Zenodo, 2019. doi: 10.5281/zenodo.3264929.

For peer review only - http://bmjopen.bmj.com/site/about/guidelines.xhtml

41 R Core Team. R: A language and environment for statistical computing [program]. R Foundation for Statistical Computing, 2021. https://www.R-project.org/.

42 Bates D, Maechler M, Bolker B, et al. Fitting linear mixed-effects models using lme4. *J Stat Softw* 2015;67:1–48 doi: 10.18637/jss.v067.i01.

43 Pinheiro J, Bates D, DebRoy S, et al. nlme: Linear and Nonlinear Mixed Effects Models. R package version 3.1-152 [program]. CRAN, 2021. https://CRAN.R-project.org/package=nlme.

44 Fox J. Effect displays in R for generalised linear models. *J Stat Softw* 2003;8:1–27.

45 Wickham H. ggplot2: Elegant Graphics for Data Analysis. New York: Springer-Verlag 2016.

46 Harrell FE Jr. Hmisc: Harrell Miscellaneous. R package version 4.6-0. [program]. CRAN, 2021. https://CRAN.R-project.org/package=Hmisc.

47 Lusambili A, Khaemba P, Chabeda S, et al. Community perspectives on maternal and newborn experiences of heat stress: a qualitative inquiry in Kilifi, Kenya. *Trop Med Int Health* 2021;26:253. doi: 10.1111/tmi.13633.

48 Oyelana O, Kamanzi J, Richter S. A critical look at exclusive breastfeeding in Africa: Through the lens of diffusion of innovation theory. *International Journal of Africa Nursing Sciences* 2021;14:100267 doi: https://doi.org/10.1016/j.ijans.2020.100267.

49 Tsegaye M, Ajema D, Shiferaw S, et al. Level of exclusive breastfeeding practice in remote and pastoralist community, Aysaita woreda, Afar, Ethiopia. *Int Breastfeed J* 2019;14:6 doi: 10.1186/s13006-019-0200-6.

50 Joseph FI, Earland J. A qualitative exploration of the sociocultural determinants of exclusive breastfeeding practices among rural mothers, North West Nigeria. *Int Breastfeed J* 2019;14:38 doi: 10.1186/s13006-019-0231-z.

51 Abas AH, Ahmed AT, Farah AE, et al. Barriers to optimal maternal and child feeding practices in pastoralist areas of Somali Region, eastern Ethiopia: A qualitative study. *Food and Nutrition Sciences* 2020;11:540-61.

52 New South Wales Ministry of Health. Babies and Children in Hot Weather. Sydney, Australia: NSW Government; 2015. https://www.health.nsw.gov.au/environment/factsheets/Pages/babies-children-hot-weather.aspx (accessed 04 August 2022).

53 Chakona G. Social circumstances and cultural beliefs influence maternal nutrition, breastfeeding and child feeding practices in South Africa. *Nutrition Journal* 2020;19:47 doi: 10.1186/s12937-020-00566-4.

54 Gatti L. Maternal perceptions of insufficient milk supply in breastfeeding. *J Nurs Scholarsh* 2008;40:355–63 doi: 10.1111/j.1547-5069.2008.00234.x.

55 Bentley GR. Hydration as a limiting factor in lactation. *Am J Hum Biol* 1998;10:151–61 doi: 10.1002/(SICI)1520-6300(1998)10:2<151::AID-AJHB2>3.0.CO;2-O.

56 Schuster RC, Butler MS, Wutich A, et al. “If there is no water, we cannot feed our children”: The far-reaching consequences of water insecurity on infant feeding practices and infant health across 16 low- and middle-income countries. *Am J Hum Biol* 2020;32:e23357 doi: 10.1002/ajhb.23357.

57 Sachdev HP, Krishna J, Puri RK. Do exclusively breast fed infants need fluid supplementation? *Indian Pediatr* 1992;29:535-40.

58 Ashraf RN, Jalil F, Aperia A, et al. Additional water is not needed for healthy breast-fed babies in a hot climate. *Acta Paediatr* 1993;82:1007–11 doi: 10.1111/j.1651-2227.1993.tb12799.x.

59 Cohen RJ, Brown KH, Rivera LL, et al. Exclusively breastfed, low birthweight term infants do not need supplemental water. *Acta Paediatr* 2000;89:550-52 doi: 10.1080/080352500750027835.

60 Bernabucci U, Basiricò L, Morera P. Impact of hot environment on colostrum and milk composition. *Cell Mol Biol* 2013;59:67–83 doi: 10.1170/T948.

61 Kobayashi K, Tsugami Y, Matsunaga K, et al. Moderate high temperature condition induces the lactation capacity of mammary epithelial cells through control of STAT3 and STAT5 signaling. *J Mammary Gland Biol Neoplasia* 2018;23:75–88 doi: 10.1007/s10911-018-9393-3.

62 Ziomkiewicz A, Babiszewska M, Apanasewicz A, et al. Psychosocial stress and cortisol stress reactivity predict breast milk composition. *Sci Rep* 2021;11:11576 doi: 10.1038/s41598-021-90980-3.

63 Caparros-Gonzalez RA, Romero-Gonzalez B, Gonzalez-Perez R, et al. Maternal and neonatal hair cortisol levels and psychological stress are associated with onset of secretory activation of human milk production. *Adv Neonatal Care* 2019;19:E11–20 doi: 10.1097/ANC.0000000000000660.

For peer review only

For peer review only - http://bmjopen.bmj.com/site/about/guidelines.xhtml

64 Cresswell JA, Ganaba R, Sarrassat S, et al. The effect of the Alive & Thrive initiative on exclusive breastfeeding in rural Burkina Faso: a repeated cross-sectional cluster randomised controlled trial. *Lancet Glob Health* 2019;7:e357–65 doi: 10.1016/S2214-109X(18)30494-7.
65 IPCC. Summary for Policymakers. In: V MassonDelmotte, P Zhai, A Pirani, et al., eds. Climate Change 2021: The Physical Science Basis Contribution of Working Group I to the Sixth Assessment Report of the Intergovernmental Panel on Climate Change. Cambridge, UK: Cambridge University Press In press.

For peer review only

For peer review only - http://bmjopen.bmj.com/site/about/guidelines.xhtml

Figure legends

Figure 1. Sampling flow chart for secondary analysis, showing number of interviews conducted with pregnant (blue) and postpartum women (green) at each interview round (T). Data from all interviews highlighted in green were included in the secondary analyses.

Figure 2. Average time spent (self-reported minutes/day) (a) breastfeeding and (b) caring for children over time, with fitted natural cubic splines of time (dashed lines). Blue shading = dry, cooler season (Nov–Feb), red shading = dry, hot season (Mar–May), green shading = rainy season (June–Oct). *N* = sample size. Data source: PopDev study. [36]

Figure 3. Scatterplots of daily mean temperature (°C) and standardised residuals from fitted trends (natural cubic splines of time) in the (a) breastfeeding and (b) childcare time-series, with locally weighted smoothing (blue line) and 95% confidence intervals (grey shading). *N* = sample size.

Figure 4. Interaction effect of daily mean temperature (°C) and infant age on time spent breastfeeding (self-reported minutes/day). *N* = sample size.

For peer review only

For peer review only - http://bmjopen.bmj.com/site/about/guidelines.xhtml

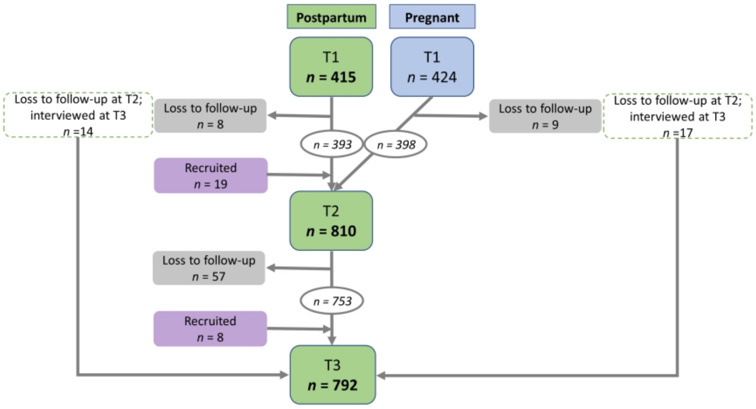

Figure 1. Sampling flow chart for secondary analysis, showing number of interviews conducted with pregnant (blue) and postpartum women (green) at each interview round (T). Data from all interviews highlighted in green were included in the secondary analyses.

338x190mm (300 x 300 DPI)

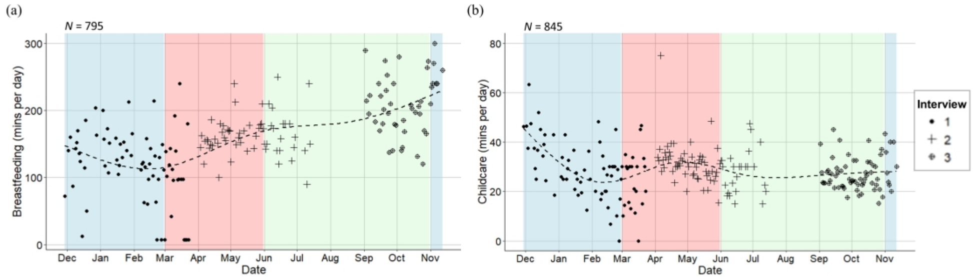

Figure 2. Average time spent (self-reported minutes/day) (a) breastfeeding and (b) caring for children over time, with fitted natural cubic splines of time (dashed lines). Blue shading = dry, cooler season (Nov–Feb), red shading = dry, hot season (Mar–May), green shading = rainy season (June–Oct). *N* = sample size. Data source: PopDev study. [37]

338x190mm (300 x 300 DPI)

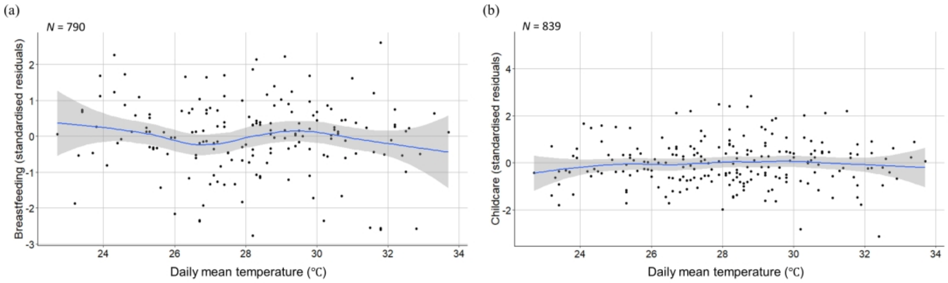

Figure 3. Scatterplots of daily mean temperature (°C) and standardised residuals from fitted trends (natural cubic splines of time) in the (a) breastfeeding and (b) childcare time-series, with locally weighted smoothing (blue line) and 95% confidence intervals (grey shading). *N* = sample size.

338x190mm (300 x 300 DPI)

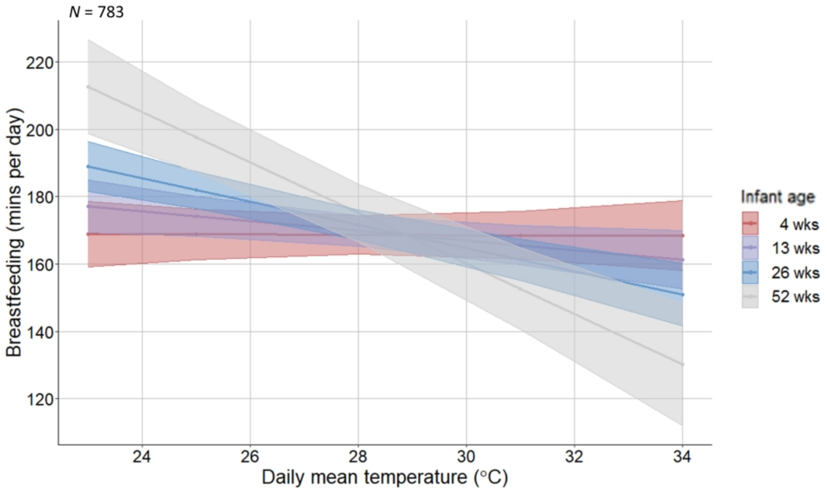

Figure 4. Interaction effect of daily mean temperature (°C) and infant age on time spent breastfeeding (self-reported minutes/day). *N* = sample size.

338x190mm (300 x 300 DPI)

SUPPLEMENTAL MATERIAL

How do high ambient temperatures affect infant feeding practices? A prospective cohort study of postpartum women in Bobo-Dioulasso, Burkina Faso

Contents

Table S1. Outcomes, exposure, potential confounders and covariates considered in all models. 2

Figure S1. Daily maximum and minimum temperatures and seasons in Bobo-Dioulasso, Burkina Faso 3

Table S2. Effect estimates, 95% confidence intervals, and *p*-values of each variable included in the multilevel linear model of temperature-breastfeeding association 4

Table S3. Effect estimates, 95% confidence intervals, and *p*-values of each variable included in the multilevel linear model of temperature-childcare association 5

References 5

For peer review only

Table S1. Outcomes, exposure, potential confounders and covariates considered in all models.

Variable	Definition	Units	Type	Model
**Outcomes**				
Breastfeeding duration	Time spent breastfeeding infants aged 4+ days	Minutes per day	Continuous	MLM (BF)
Exclusive breastfeeding	No liquids other than breast milk given in past 24h (infants aged <6 months)	Yes, No	Binary	Logistic (EBF)
Supplementary feeding	Any liquid other than breast milk given in past 24h (infants aged 6-12 months)	Yes, No	Binary	Logistic (SF)
Childcare duration	Time spent caring for children (bathing/dressing, playing/watching, tending to when unwell, etc.)	Minutes per day	Continuous	MLM (CH)
**Exposure**				
Daily mean temperature	Mean temperature in Bobo-Dioulasso on day before interview	°C	Continuous	All
**Confounders**				
Season/time trends	Seasonal patterns (unrelated to temperature) and long-term trends	Month of interview	Categorical	All
Interview round	Interview (baseline, 3 months post-baseline, 9 months post-baseline)	1, 2, 3	Categorical	BF, CH
Infant age	Date of interview – Date of delivery	Weeks	Continuous	All
Income-generating work	Time spent on professional or educational activities	Minutes per day	Continuous	All
Domestic work	Time spent on domestic activities (excluding family care)	Minutes per day	Continuous	BF, EBF, SF
**Covariates**				
Number born	Singleton or multiple birth	1, 2	Categorical	All
Gravidity	Total number of pregnancies, including current	1, 2–5, 6+ pregnancies	Categorical	All
Maternal age	Age of mother at baseline	≤ 19, 20–34, ≥ 35 years	Categorical	All
Living arrangements	Mothers' living arrangements with partner at each interview	With partner full-time, With partner periodically, Not with partner, Not in a relationship	Categorical	CH
Residential area	Mother's area of residence	Urban, Rural	Categorical	All
Roofing materials	Roofing materials of mother's house	Natural, Rudimentary, Contemporary	Categorical	All
**Interactions**				
*Temperature * Residential area*	Daily mean temperature and mother's area of residence	°C * Urban/Rural	Interaction	All
*Temperature * Infant age*	Daily mean temperature and infant age	°C * Weeks	Interaction	All
*Temperature * Roofing materials*	Daily mean temperature and roofing materials of mother's house	°C * Natural/Rudimentary/ Contemporary	Interaction	All

MLM = Multilevel model. BF = Breastfeeding duration. CH = Childcare duration. SF = Supplementary feeding. EBF = Exclusive breastfeeding.

For peer review only - http://bmjopen.bmj.com/site/about/guidelines.xhtml

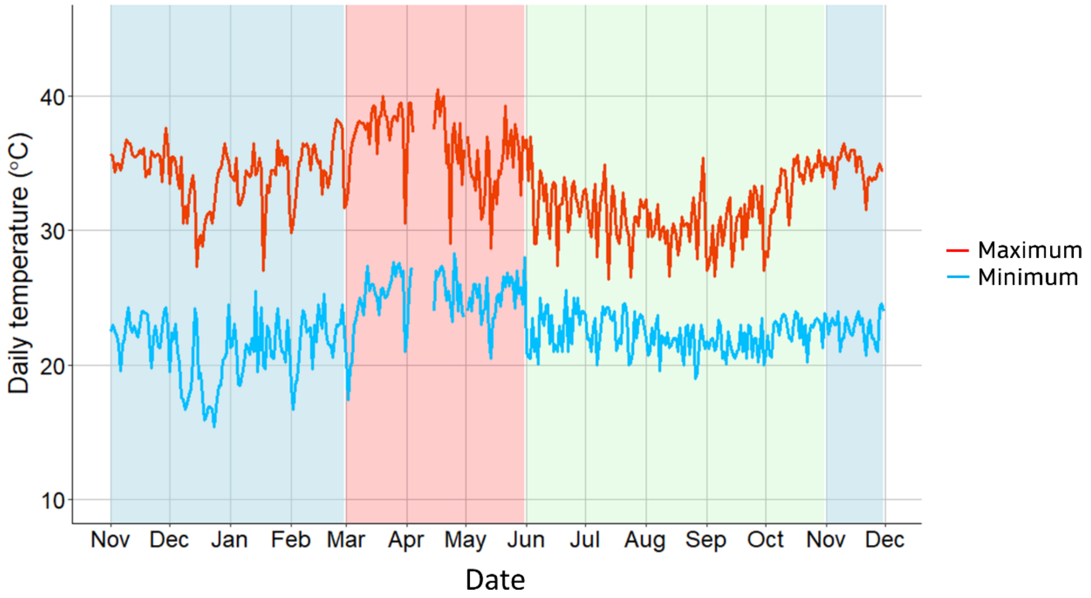

Figure S1. Daily maximum and minimum temperatures and seasons in Bobo-Dioulasso, Burkina Faso, between 1st Nov 2013 and 30th Nov 2014. Blue = dry, cooler season (November–February), red = dry, hot season (March–May), green = rainy season (June–October). Data source: TuTiempo.net [1].

For peer review only - http://bmjopen.bmj.com/site/about/guidelines.xhtml

Table S2. Effect estimates, 95% confidence intervals, and *p*-values of each variable included in the autoregressive multilevel linear model for the exposure-response association between temperature and breastfeeding duration (minutes/day).

**Variable**	**Estimate**	**95% CI**		***p*-value**
Daily mean temperature (°C)	-2.29	-4.63	0.04	0.05
Interview round (reference: 1)				
2	32.01	-52.53	116.54	0.46
3	135.46	59.35	211.57	<0.001
Month of data collection (reference: January)				
February	-34.16	-61.25	-7.07	0.01
March	-61.79	-89.14	-34.45	<0.001
April	-7.70	-95.20	79.79	0.86
May	-22.75	-106.68	61.18	0.59
June	-37.01	-122.13	48.12	0.39
July	-37.57	-135.16	60.02	0.45
September	-89.72	-167.34	-12.09	0.02
October	-102.25	-178.37	-26.14	<0.01
November	-66.34	-136.35	3.66	0.06
December	-13.72	-35.25	7.81	0.21
Singleton/multiple birth (reference: singleton)				
Multiple birth	70.46	48.43	92.49	< 0.001
Residential area (reference: rural)				
Urban	18.26	-5.76	42.29	0.13
Income-generating activities (minutes/day)	0.02	0.00	0.04	0.02

For peer review only

Table S3. Effect estimates, 95% confidence intervals, and *p*-values of each variable included in the autoregressive multilevel linear model for the exposure-response association between temperature and childcare duration (minutes/day).

Variable	Estimate	95% CI		*p*-value
Daily mean temperature (°C)	0.65	0.06	1.24	0.03
Interview round (reference: 1)				
2	-8.09	-34.64	18.47	0.55
3	-15.25	-36.13	5.63	0.15
Month of data collection (reference: January)				
February	-1.75	-7.64	4.13	0.56
March	-4.36	-10.33	1.60	0.15
April	9.96	-17.18	37.09	0.47
May	8.99	-17.90	35.87	0.51
June	8.32	-18.84	35.48	0.55
July	3.48	-25.75	32.71	0.82
September	15.08	-5.95	36.10	0.16
October	12.88	-8.13	33.90	0.23
November	16.52	-2.63	35.66	0.09
December	14.34	8.75	19.92	< 0.001
Singleton/multiple birth (reference: singleton)				
Multiple birth	17.43	11.53	23.33	< 0.001
Infant age (weeks)	0.09	-0.04	0.21	0.17
Maternal age (reference: 20-34 years)				
< 19 years	-5.68	-8.68	-2.69	< 0.001
≥ 35 years	0.29	-2.39	2.97	0.83
Income-generating activities (minutes/day)	-0.01	-0.01	-0.00	0.04
Living arrangements (reference: Lives with partner full-time)				
Lives with partner periodically	-1.91	-5.73	1.92	0.33
Does not live with partner	-2.57	-7.42	2.29	0.30
Not in a relationship	-8.68	-12.47	-4.89	< 0.001

References

1 TuTiempo. Clima Bobo-Dioulasso. Datos climáticos: 1973 - 2021. https://www.tutiempo.net/clima/ws-655100.html.

For peer review only - http://bmjopen.bmj.com/site/about/guidelines.xhtml

STROBE Statement—Checklist of items that should be included in reports of ***cohort studies***

	Item No	Recommendation	Page No
**Title and abstract**	1	(*a*) Indicate the study's design with a commonly used term in the title or the abstract	1
		(*b*) Provide in the abstract an informative and balanced summary of what was done and what was found	3-4
**Introduction**			
Background/rationale	2	Explain the scientific background and rationale for the investigation being reported	6-8
Objectives	3	State specific objectives, including any prespecified hypotheses	8
**Methods**			
Study design	4	Present key elements of study design early in the paper	8
Setting	5	Describe the setting, locations, and relevant dates, including periods of recruitment, exposure, follow-up, and data collection	9-10
Participants	6	(*a*) Give the eligibility criteria, and the sources and methods of selection of participants. Describe methods of follow-up	8-9
		(*b*) For matched studies, give matching criteria and number of exposed and unexposed	N/A
Variables	7	Clearly define all outcomes, exposures, predictors, potential confounders, and effect modifiers. Give diagnostic criteria, if applicable	12, Table S1
Data sources/ measurement	8*	For each variable of interest, give sources of data and details of methods of assessment (measurement). Describe comparability of assessment methods if there is more than one group	9-11
Bias	9	Describe any efforts to address potential sources of bias	9, 10-11,13
Study size	10	Explain how the study size was arrived at	N/A
Quantitative variables	11	Explain how quantitative variables were handled in the analyses. If applicable, describe which groupings were chosen and why	12-13, 14
Statistical methods	12	(*a*) Describe all statistical methods, including those used to control for confounding	12-15
		(*b*) Describe any methods used to examine subgroups and interactions	14
		(*c*) Explain how missing data were addressed	14
		(*d*) If applicable, explain how loss to follow-up was addressed	14
		(*e*) Describe any sensitivity analyses	14
**Results**			
Participants	13*	(a) Report numbers of individuals at each stage of study—eg numbers potentially eligible, examined for eligibility, confirmed eligible, included in the study, completing follow-up, and analysed.	Figure 1, Table 1, 18, 19
		(b) Give reasons for non-participation at each stage	18
		(c) Consider use of a flow diagram	Figure 1
Descriptive data	14*	(a) Give characteristics of study participants (eg demographic, clinical, social) and information on exposures and potential confounders	Table 1, 16-18, Figure S1
		(b) Indicate number of participants with missing data for each variable of interest	Table 1
		(c) Summarise follow-up time (eg, average and total amount)	18

For peer review only - http://bmjopen.bmj.com/site/about/guidelines.xhtml

For peer review only

Outcome data	15*	Report numbers of outcome events or summary measures over time	Table 1, Figure 2, 18
Main results	16	(*a*) Give unadjusted estimates and, if applicable, confounder-adjusted estimates and their precision (eg, 95% confidence interval). Make clear which confounders were adjusted for and why they were included	18-19, Table S2, Table S3
		(*b*) Report category boundaries when continuous variables were categorized	N/A
		(*c*) If relevant, consider translating estimates of relative risk into absolute risk for a meaningful time period	N/A
Other analyses	17	Report other analyses done—eg analyses of subgroups and interactions, and sensitivity analyses	19, 20
**Discussion**			
Key results	18	Summarise key results with reference to study objectives	20
Limitations	19	Discuss limitations of the study, taking into account sources of potential bias or imprecision. Discuss both direction and magnitude of any potential bias	22-23
Interpretation	20	Give a cautious overall interpretation of results considering objectives, limitations, multiplicity of analyses, results from similar studies, and other relevant evidence	20-24
Generalisability	21	Discuss the generalisability (external validity) of the study results	23
**Other information**			
Funding	22	Give the source of funding and the role of the funders for the present study and, if applicable, for the original study on which the present article is based	25

*Give information separately for exposed and unexposed groups.

Note: An Explanation and Elaboration article discusses each checklist item and gives methodological background and published examples of transparent reporting. The STROBE checklist is best used in conjunction with this article (freely available on the Web sites of PLoS Medicine at http://www.plosmedicine.org/, Annals of Internal Medicine at http://www.annals.org/, and Epidemiology at http://www.epidem.com/). Information on the STROBE Initiative is available at http://www.strobe-statement.org.

For peer review only
